# Multi-omics reveals the phyllosphere microbial community and material transformations in cigars

**DOI:** 10.3389/fmicb.2024.1436382

**Published:** 2024-07-31

**Authors:** Xiaoyu Wang, Shuai Yang, Qiang Gao, Youqing Dai, Lei Tian, Liang Wen, Honghao Yan, Long Yang, Xin Hou, Peng Liu, Li Zhang

**Affiliations:** ^1^College of Plant Protection, Shandong Agricultural University, Tai’an, China; ^2^Yuxi Zhongyan Tobacco Seed Co., Ltd., Yu’xi, China; ^3^Shandong Linyi Tobacco Co., Ltd., Lin’yi, China; ^4^Cigar Operating Centre of China Tobacco Shandong Industrial Co., Ltd., Ji’nan, China

**Keywords:** cigar tobacco leaf, fermentation, phyllosphere microbial community, chemical composition, multi-omics, metabolomics

## Abstract

The quality of fermented plant leaves is closely related to the interleaf microorganisms and their metabolic activities. In this experiment, a multi-omics analysis was applied to investigate the link between the structural composition of the phyllosphere microbial community and the main metabolites during the fermentation process. It was found that the whole fermentation process of cigar leaves could be divided into three stages, in which the Mid-Stage was the most active period of microbial metabolic activities and occupied an important position. *Staphylococcus, Brevundimonas, Acinetobacter, Brevibacterium, Pantoea, Aspergillus, Wallemia, Meyerozyma, Sampaiozyma, Adosporium* and *Trichomonascus* played important roles in this fermentation. *Staphylococcus* and *Aspergillus* are the microorganisms that play an important role in the fermentation process. *Staphylococcus* were strongly correlated with lipids and amino acids, despite its low abundance, *Stenotrophomonas* is importantly associated with terpene and plays a significant role throughout the process. It is worth noting that Wapper exists more characteristic fungal genera than Filler and is more rapid in fermentation progress, which implies that the details of the fermentation process should be adjusted appropriately to ensure stable quality when faced with plant leaves of different genotypes. This experiment explored the relationship between metabolites and microorganisms, and provided a theoretical basis for further optimizing the fermentation process of plant leaves and developing techniques to improve product quality. Biomarker is mostly present in the pre-fermentation phase, but the mid-fermentation phase is the most important part of the process.

## Graphical abstract

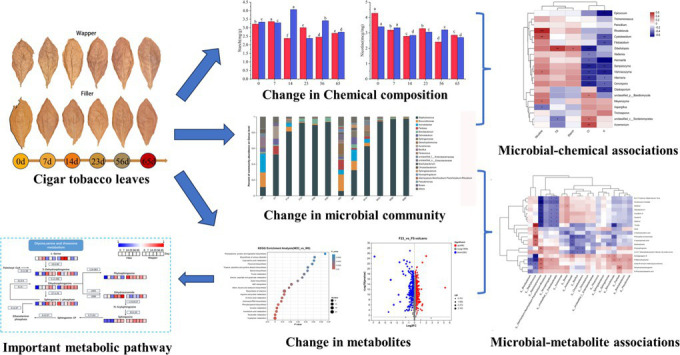

## Highlights

- Biomarker is mostly present in the pre-fermentation phase, but the mid-fermentation phase is the most important part of the process.
- *Staphylococcus* is the most important bacterial genus, and *Stenotrophomonas*, although less abundant, still plays an important role.
- Sphingolipid metabolites are active during fermentation and may have an important effect on fermentation quality.

## 1 Introduction

Fermentation, a venerable food preservation and processing technique, holds a pivotal position in the manufacture of diverse bean, meat, and plant-based products (Leeuwendaal et al., 2022). As demonstrated in numerous studies (Chupeerach et al., 2021; Liu et al., 2022; Leroy et al., 2023), fermentation not only enhances the unique and diverse flavor profiles of these products but also elevates their overall quality (Wen et al., 2023). Fundamentally, fermentation entails the intricate biochemical transformation of substrates, driven by microbial metabolic activities and enzymatic reactions. These reactions facilitate the breakdown of macromolecules, such as starch, proteins, and lipids, into smaller organic molecules, including reducing sugars, aldehydes, and ketones, which ultimately contribute to the complex aroma, taste, and flavor profiles of the final product (van Wyk, 2024).

In the context of fermented sauerkraut, fatty acids undergo enzymatic degradation, yielding a plethora of volatile aldehydes that significantly contribute to the food’s refreshing aroma (Li et al., 2022). During the terminal stages of black tea fermentation, enzymatic reactions facilitate the formation of an array of ketones, which impart the tea’s characteristic floral and woody aroma (Hu et al., 2022). In the brewing process of yellow wine, lactic acid bacteria modulate the ethyl lactate content through intricate enzymatic reactions, resulting in the production of compounds such as isoamyl butyrate, ethyl caprate, ethyl butyrate, and ethyl phenylethylacetate, which enrich the mellowness of the wine’s flavor and significantly enhance its overall quality (Guo et al., 2024). Terpenoids play a pivotal role in shaping the aroma of tobacco, with neophytadiene serving as a defining characteristic of tobacco flavor (Zhang et al., 2023). In cigars, esters, ketones (e.g., lycopene), and aldehydes (2,4-dimethylbenzaldehyde) contribute uniquely to their flavor profiles (Gaspar et al., 2019; Zheng et al., 2022). However, excessive starch content can lead to a burnt odor during cigar combustion, while high nicotine levels may impart an irritating and harsh smoke (Zhao et al., 2009, 2012). To ensure the consistent maintenance of fermentation product quality, a comprehensive understanding of the metabolites during the fermentation process is imperative. While current research has primarily focused on the impact of chemical composition on plant leaf fermentation product quality, there is a need for more in-depth studies exploring the diverse range of metabolites that emerge during fermentation.

As a distinct tobacco product, the quality of cigars is profoundly shaped by the fermentation process, a complex biotransformation reliant on the synergistic activities of a diverse array of microorganisms (Jia et al., 2023). The fermentation of cigar tobacco leaves (CTLs) is primarily governed by interleaf microbiota, though their community structure exhibits significant variations influenced by geographical, climatic, and varietal factors (Ren et al., 2023), thereby contributing to diverse cigar flavor profiles and quality attributes. *Staphylococcus* is integral to carbohydrate catabolism, amino acid transformation, protein hydrolysis, and lipolysis, mediating the degradation of branched-chain amino acids into methyl-branched-chain alcohols, aldehydes, carboxylic acids, and their corresponding esters (Zhao et al., 2009). *Pseudomonas* facilitate the enzymatic breakdown of proteins, enriching the amino acid pool, which significantly contributes to flavor development (Dzialo et al., 2017; Murata, 2021). Furthermore, they degrade cellular components such as cellulose, pectin, starch, and lignin in tobacco leaves, releasing flavor-active small molecules including esters, alcohols, and aldehydes (Banožić et al., 2020; Liu et al., 2021; Wang et al., 2022). *Bacillus* play a pivotal role in degrading macromolecules like carotenoids, yielding significant small-molecule aromatic compounds such as megacolumnar trienones, isophorones, and 2,6,6-trimethyl-2-cyclohexene-1,4-diones (Dai et al., 2020). These bacteria also secrete enzymes that degrade starch, proteins, and cellulose in tobacco, further contributing to the complex flavor profile (Pen et al., 1992; Li et al., 2006; Singh et al., 2017). The fungal community within cigar leaves exhibits a relatively simpler structure compared to bacteria, with *Aspergillus* often emerging as the dominant species. However, *Alternaria* and *Wallemia* are also prevalent and play equally crucial roles in the fermentation process. *Aspergillus* species exhibit robust activity in the transformation of saccharide compounds (Jia et al., 2023), demonstrating a strong capability to decompose starch and organic nitrogen under conditions of sufficient oxygen and optimal humidity (Yang et al., 2022).

Multiomics analysis has gained widespread application in the realm of fermented plant products, encompassing olives, tea, and vegetables (Rizo et al., 2020; Vaccalluzzo et al., 2020; Hu et al., 2022). It holds a pivotal role in investigating fermentation processes and mechanisms underlying flavor development. Recently, in examining the interplay between CTLs fermentation and the composition of interfollicular microbial communities (Wu et al., 2021; Zheng et al., 2022). However, our comprehension of the structural dynamics of the foliar microbiome and the specific mechanisms driving aroma formation during fermentation remains elusive. This work employed high-throughput sequencing technology, coupled with a comprehensive analysis of microbiomics and metabolomics, to identify functional microorganisms and crucial metabolites involved in the fermentation process. Furthermore, we delved into the correlation between microbial communities and metabolite alterations, providing both theoretical and technical foundations for optimizing plant leaf fermentation-related production processes.

## 2 Materials and methods

### 2.1 Source of cigar tobacco headings

Two premium cigar varieties were selected from Xishuangbanna, Yunnan Province, China: YX1 (packed) and YX6 (filled). Unfermented cigar samples were collected at the end of the drying treatment (0d), while five nodes were selected throughout the stacking and fermentation process, each of which was turned over after the temperature and humidity reached the preset conditions (7d, 14d, 22d, 56d, and 65d), labeled W0, W7, W14, W22, W56, and W65, respectively; and F0, F7, F14, F22, and F56 each time. At each turning of the pile, 3 pieces of cigar leaves were taken out from the upper, middle and lower layers, from which 6 pieces of cigar leaves with the same appearance and quality were selected, and about 10 grams of the same portion of the leaves were cut off with sterilized scissors, put into centrifugal tubes, labeled, and stored in a refrigerator at −80°C. The leaves were then stored at −80°C for a short time.

### 2.2 DNA extraction and Illumina MiSeq sequencing

Three replicate samples of 5 g each were taken from each group, and the extract was obtained by adding 250 mL 1% PBS buffer. Microbes on fermented leaves were collected by shaking the flasks and centrifuging the eluent. The total genomic DNA of the microbial community was extracted according to the instructions of the E.Z.N.A.^®^ soil DNA kit (Omega Bio-tek, Norcross, GA, United States), and the quality of the extracted genomic DNA was checked by agarose gel electrophoresis with 1% agarose, and the concentration and purity of the DNA were determined by NanoDrop2000 (Thermo Scientific, United States). The extracted DNA was used as a template, and upstream primer 338F (5’-ACTCCTACGGGGAGGCAGCAG-3′) and downstream primer 806R (5’-GGACTACHVGGGGGGG), which carried the Barcode sequence, were used as templates. (GGACTACHVGGGTWTCTAAT-3′) were used to amplify the V3–V4 variable region of the 16S rRNA gene by PCR, and the PCR products were recovered on a 2% agarose gel, and purified using the PCR Clean-Up Kit (PCR Clean-Up Kit, China). The recovered products were purified using the DNA Gel Purification Kit (PCR Clean-Up Kit, Qubit 4.0, Thermo Fisher Scientific, United States) and quantified using Qubit 4.0. The purified PCR products were library-constructed using NEXTFLEX Rapid DNA-Seq Kit and sequenced using Illumina PE300/PE250 platform.

### 2.3 Chemical composition determination

Each group of three replicate samples was dried in an electric blast dryer at 40°C until the leaves were shaken with a breaking sound, easily broken by hand squeezing and ground for 100 s, passed through a 60-mesh sieve, added 5% acetic acid and sealed with a sealing film, extracted by shaking for 30 min on an oscillator and filtered through a qualitative filter paper to collect the remaining filtrate in addition to the first 5 mL, and determined the chlorine ion content of the tobacco by using a continuous flow analyzer (AA3 Germany) in accordance with the national standard (YC/ T162-2011). The total sugar, starch, nicotine, chloride and potassium contents of CTLs were determined by continuous flow analysis (AA3, Germany) according to the national standard (YC/T162-2011).

### 2.4 Metabolom analysis

The LC–MS/MS analysis of sample was conducted on a Thermo UHPLC-Q Exactive HF-X system equipped with an ACQUITY HSS T3 column (100 mm × 2.1 mm i.d., 1.8 μm; Waters, United States) at Majorbio Bio-Pharm Technology Co., Ltd. (Shanghai, China). The mobile phases consisted of 0.1% formic acid in water: acetonitrile (95:5, v/v) (solvent A) and 0.1% formic acid in acetonitrile: isopropanol: water (47.5:47.5, v/v) (solvent B). MS conditions: The flow rate was 0.40 mL/min and the column temperature was 40°C. The injection volume was 3 μL. The mass spectrometric data were collected using a Thermo UHPLC-Q Exactive HF-X Mass Spectrometer equipped with an electrospray ionization (ESI) source operating in positive mode and negative mode. The optimal conditions were set as followed: Aux gas heating temperature at 425°C; Capillary temp at 325°C; sheath gas flow rate at 50 psi; Aux gas flow rate at 13 psi; ion-spray voltage floating (ISVF) at −3,500 V in negative mode and 3,500 V in positive mode, respectively; Normalized collision energy, 20–40–60 eV rolling for MS/MS. Full MS resolution was 60,000, and MS/MS resolution was 7,500. Data acquisition was performed with the Data Dependent Acquisition (DDA) mode. The detection was carried out over a mass range of 70–1,050 m/z.

### 2.5 Statistical analysis

Alpha diversity knowledge Chao 1, Shannon index, etc. were calculated using mothur software,^1^ and the Wilxocon rank sum test was used for the analysis of intergroup differences in Alpha diversity; the differences in microbial community structure between the sample groups were analyzed using the bray-curtis-based distance algorithm based PCoA analysis (principal coordinate analysis) to test the similarity of microbial community structure between samples, and combined with the PERMANOVA nonparametric test to analyze whether the differences in microbial community structure between sample groups were significant or not; with LEfSe analysis (Linear discriminant analysis Effect Size) (Segata et al., 2011)^2^ (LDA > 2, *p* < 0.05) to identify bacterial taxa with significant differences in abundance from phylum to genus level among different groups. Distance-based redundancy analysis (db-RDA) was used to investigate the effects of physicochemical indicators on microbial community structure. Linear regression analysis was used to assess the effect of the main soil physicochemical/clinical indicators identified in the db-RDA analysis on the microbial Alpha diversity index. Species were selected for correlation network graph analysis based on spearman correlation |r| > 0.6 *p* < 0.05 (Barberán et al., 2012).

The UHPLC–MS raw data were converted into the common format by Progenesis QI software (Waters, Milford, United States) through baseline filtering, peak identification, peak integral, retention time correction, and peak alignment. Then, the data matrix containing sample names, m/z, retention time and peak intensities was exported for further analyses. At the same time, the metabolites were identified by searching database, and the main databases were the HMDB^3^, Metlin^4^ and the self-compiled Majorbio Database (MJDB) of Majorbio Biotechnology Co., Ltd. (Shanghai, China). The data matrix obtained by searching database was uploaded to the Majorbio cloud platform^5^ for data analysis. Firstly, the data matrix was pre-processed, as follows: At least 80% of the metabolic features detected in any set of samples were retained. After filtering, the minimum value in the data matrix was selected to fill the missing value and each metabolic signature was normalized to the sum. To reduce the errors caused by sample preparation and instrument instability, the response intensities of the sample mass spectrometry peaks were normalized using the sum normalization method, to obtain the normalized data matrix. Meanwhile, the variables of QC samples with relative standard deviation (RSD) > 30% were excluded and log10 logarithmicized, to obtain the final data matrix for subsequent analysis.

## 3 Results

### 3.1 Analysis of microbial community

1,053 bacterial OTUs and 1,420 fungal OTUs were obtained from all CTLs, and plot the dilution curve of the sample by the sobs value of the respective microbial communities. The adequacy of the sequencing data was determined by observing whether the curve reached a plateau. As the amount of sequencing increased, the sobs index, representing the number of observed OTUs, ceased to rise and gradually flattened out, indicating that the sequencing depth was sufficient to capture most of the microbial diversity in the samples. The dilution curves gradually approached a plateau with increasing sample volume, revealing more intricate dynamics in fungal diversity compared to bacteria.

**Figure 1:**
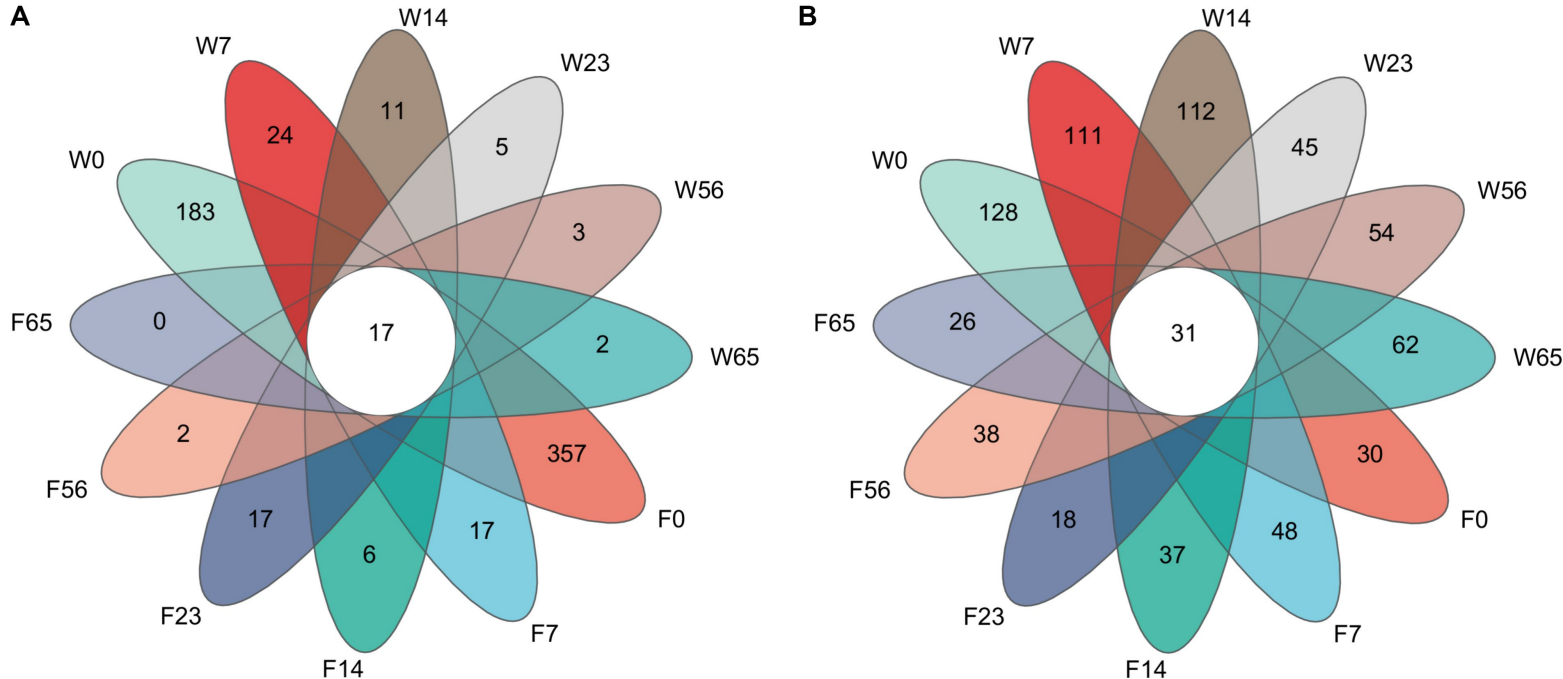
Unique and shared OTUs in fermentation processes. **(A)** bacteria, **(B)** fungi.

A comparative analysis of the 1,053 bacterial OTU representatives (Figure 1) revealed 17 shared OTUs among the samples. Among them, F0 had the highest number of unique OTUs (357), followed by W0 (183). The number of unique OTUs exhibited a sharp decline at 7 days and then a gradual decrease until the end of fermentation (65 days), where F65 no longer possessed any unique OTUs, while W65 still maintained 2 unique OTUs. In contrast, the comparative analysis of the 1,420 fungal OTU representatives yielded 31 shared OTUs, with W0 having the highest number of unique OTUs (128), followed by W14 (112). The number of unique OTUs in the Wapper showed a decreasing and then an increasing trend, and at the critical point of 23d, the number of unique OTUs in the fungal community showed a cliff-like decline similar to that in the bacterial community (Lee et al., 2015) and a slow upward trend in subsequent fermentations. In the Filler, the number of unique OTUs showed fluctuating changes and remained in a relatively stable state throughout the fermentation process, with little difference between pre-fermentation (F0:30) and post-fermentation (F65:26).

### 3.2 Diversity and abundance

**Figure 2:**
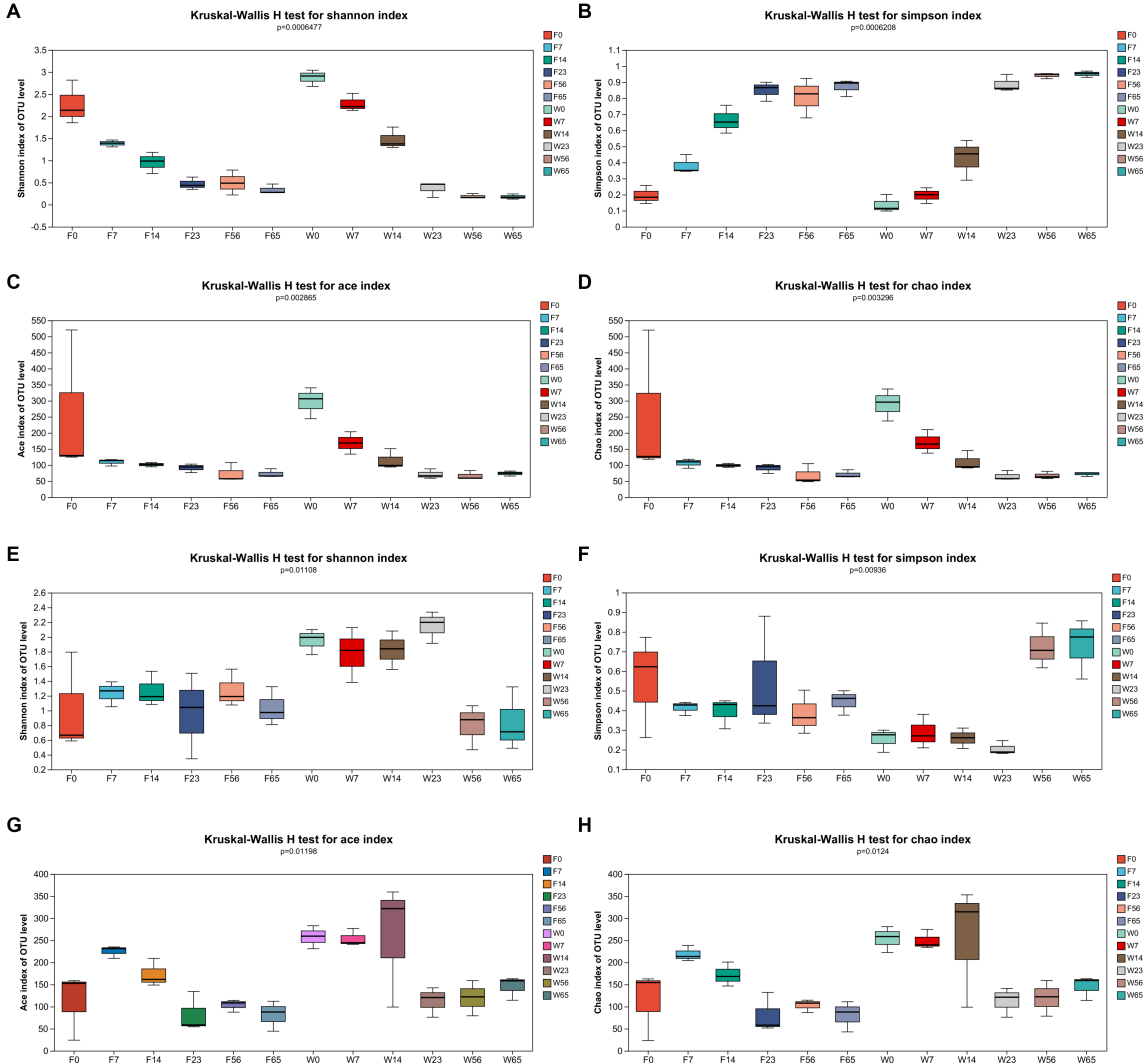
α-diversity of bacterial and fungal OTUs. Bacteria: **(A)** Shannon’s index, **(B)** Simpson’s index, **(C)** Ace’s index, and **(D)** Chao’s index. Fungi: **(E)** Shannon’s index, **(F)** Simpson’s index, **(G)** Ace’s index, and **(H)** Chao’s index.

Community richness (ACE and Chao indices) and diversity (Simpson and Shannon indices) were used to reflect the α-diversity of the CTLs microbiota and tested for intergroup differences in indices (Figure 2).

In the context of bacterial communities, as the fermentation process progressed from 0d to 65d, the Shannon index of the CTLs exhibited a pronounced declining pattern, which was corroborated by a significant rise in the Simpson index. Additionally, the ace and chao indices displayed a notable increasing trend throughout the fermentation, indicating a congruence with the microbial community diversity that aligned with the progression of the fermentation process.

Concerning fungal communities, the Shannon index of Filler exhibited a relatively stable fluctuation throughout the fermentation, although a significant decrement was observed at the endpoint compared to the initial 0d. Conversely, Wapper displayed an initial increase, peaking at 23d, followed by a decline, particularly during the late fermentation period (56d, 65d). The Simpson index of Filler showed a marked decrease in the late fermentation (56d, 65d), while Wapper exhibited a significant increase at 23d, contrasting with the Shannon index. This divergence in trends between the Shannon and Simpson indices for the Wapper samples lends credence to the data. The Ace and Chao indices exhibited a trend of initially increasing and subsequently decreasing throughout the entire fermentation.

### 3.3 Dynamics of microbial communities

**Figure 3:**
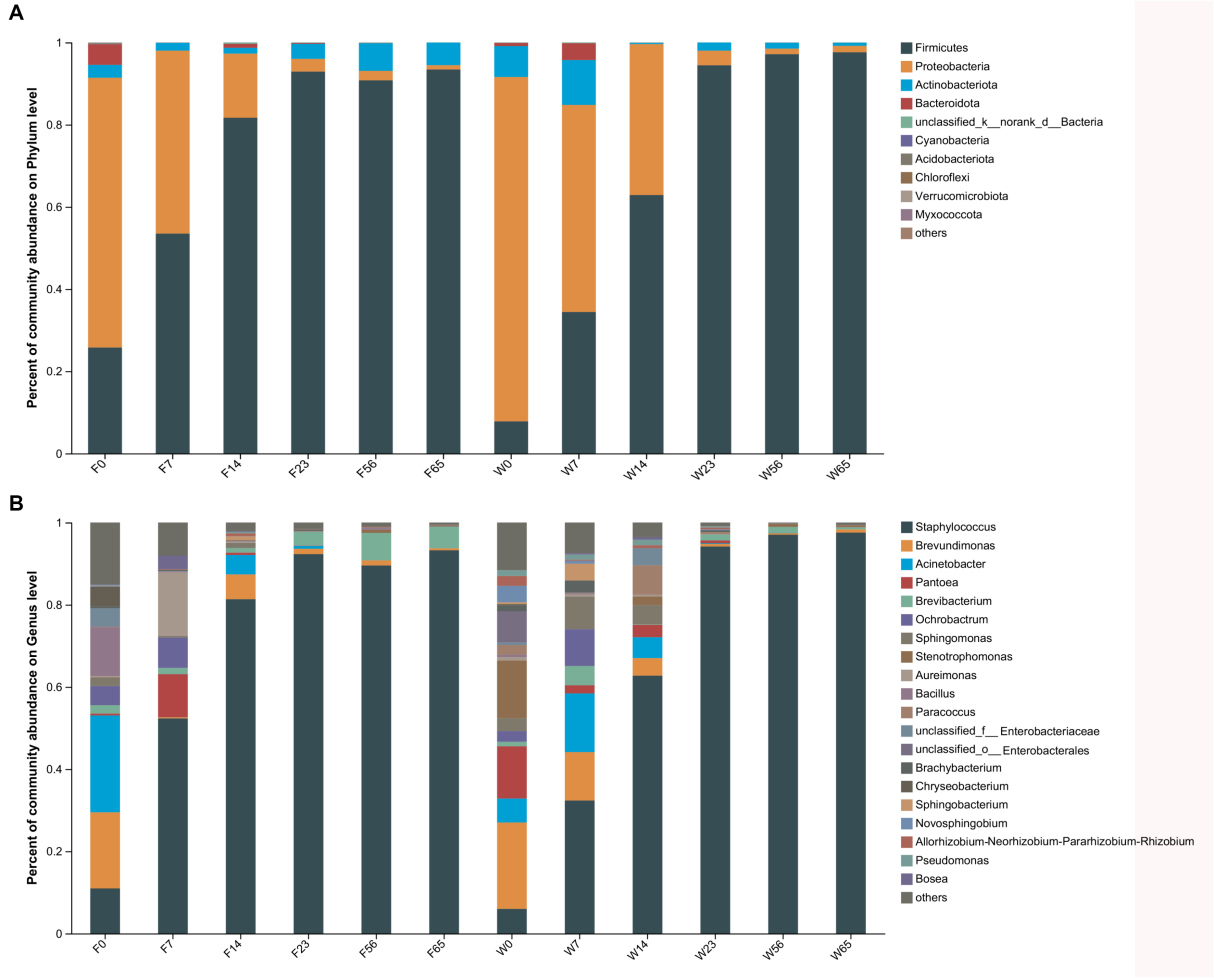
The distribution of the microbial community in CTLs at various stages of fermentation. Bacteria at the phylum level **(A)** and genus level **(B)**.

**Figure 4:**
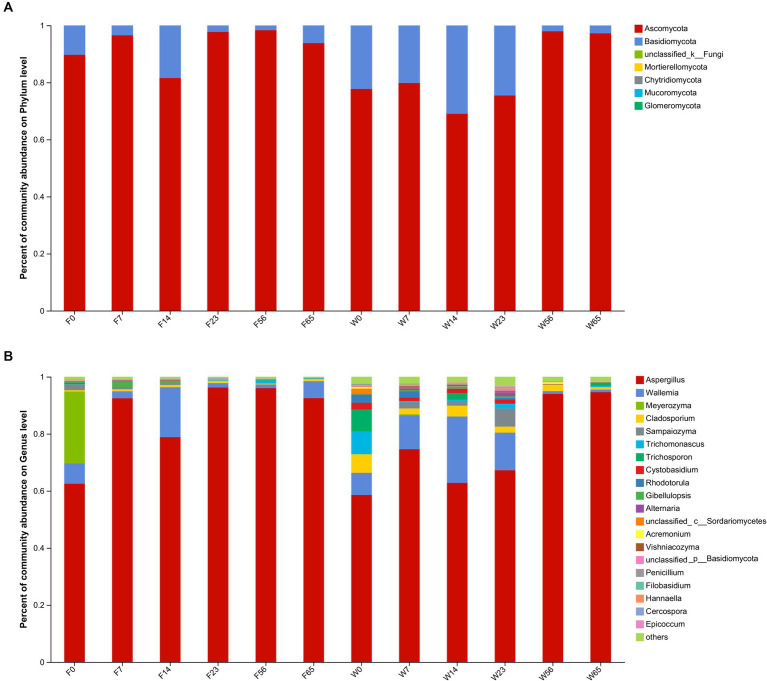
The distribution of the microbial community in CTLs at various stages of fermentation. Fungi at the phylum level **(A)** and genus level **(B)**.

The alterations in microbiota composition during cigar stack fermentation were examined through 16S and ITS amplicon sequencing. For subsequent analyses, the bacterial and fungal phyla displaying the top 10 relative abundances, as well as the bacterial and fungal genera with the top 20 relative abundances, were selected for in-depth evaluation (Figures 3, 4). Notably, the bacterial phyla *Firmicutes* (7.81–97.61%), *Proteobacteria* (1.34–83.80%) and *Actinobacteria* (0.27–10.91%). exhibited significant variations in abundance during the fermentation process. Specifically, *Firmicutes* exhibited a lower abundance initially (0d, 7d), gradually increased as fermentation progressed, and ultimately achieved an absolute dominant position towards the end of the fermentation period (56d, 65d). *Proteobacteria* displayed a high abundance in the Initial-Stages, but its abundance gradually declined as fermentation progressed. Conversely, *Actinobacteria* accounted for a relatively low percentage throughout the fermentation process, with its abundance exhibiting a trend of initial decrease followed by an increase. At the bacterial phylum level, the abundance of each dominant phylum stabilized towards the Late-Stages of fermentation.

Among the bacterial genera, *Staphylococcus* (5.99–97.54%) emerged as the most abundant, followed by *Brevundimonas*, *Staphylococcus* (distinct from the previous mention), and *Pantoea*. *Staphylococcus* initially exhibited a low abundance (0d) but gradually increased as fermentation progressed, ultimately dominating the microbial community. Conversely, *Brevundimonas*, *Brevibacterium*, and *Pantoea* displayed higher abundances initially, but gradually declined as fermentation progressed. The trend of *Brevibacterium* during fermentation was more complicated, showing a decreasing and then increasing trend in Filler, and a continuous decreasing trend in Wapper, but the abundance all stabilized at the Late-Stage (56d, 65d); in previous studies, *Bacillus* also played an important role in the fermentation process, and *Brevibacterium* possessed a high abundance in F0, but declined dramatically afterward.

During the fermentation process, *Ascomycota* exhibited the most prominent abundance (69.04–98.32%), whereas *Basidiomycota* accounted for a comparatively lower proportion, spanning (1.91–30.89%). They cumulatively comprising over 99% of the fungal community throughout the fermentation. *Ascomycota* exhibited a distinct pattern of initial increase followed by a subsequent decrease during the fermentation, whereas *Basidiomycota* exhibited an inverse trend, consonant with the observed dynamics of α-diversity.

Within the fungal genus, *Aspergillus* dominated the fermentation process (58.55 to 96.25%), followed by *Wallemia* (0.95 to 23.24%). *Aspergillus* displayed a pronounced increase in abundance during the Initial-Stages (0d, 7d), a subsequent decrease in the mid-fermentation period (14d, 23d), and a significant resurgence in the Late-Stage (56d, 65d). Conversely, *Wallemia* exhibited a trend of increasing abundance followed by a decrease. Notably, *Meyerozyma*, a yeast species renowned for its aroma-producing capabilities, possessed a substantial abundance in the Initial-stages but ceased to be detectable in the Mid-Stage, likely due to its role in contributing significant aroma in the Initial-Stage. *Sampaiozyma*, *Cladosporium* and *Trichomonascus*, although less abundant, were commonly present during fermentation and may play an important role in the fermentation of cigar tobacco.

### 3.4 Clustering analysis of microbial communities

**Figure 5:**
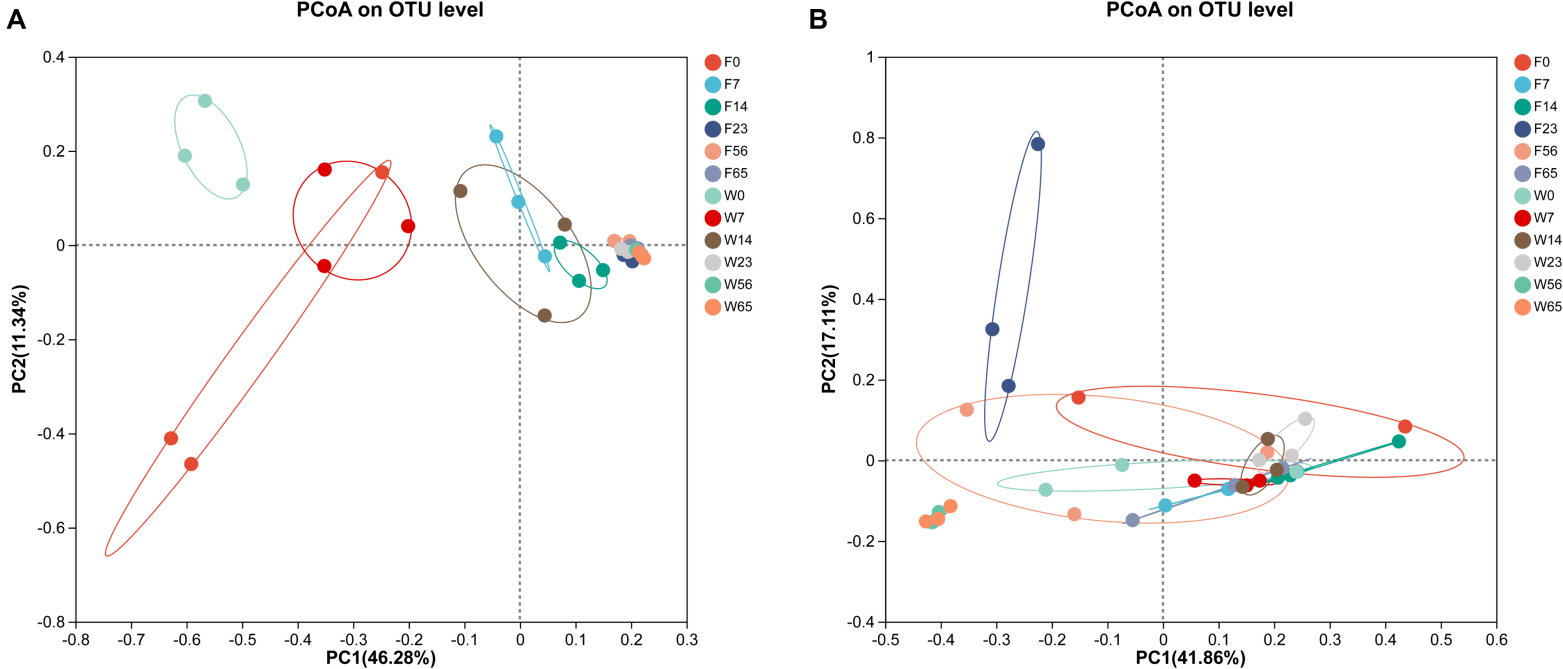
Principal co-ordinates analysis on genus level with cigar bacteria **(A)** and fungi **(B)**.

In order to more fully illustrate the differences in microbial communities among samples at different stages, cluster analysis was performed. The PCoA plot was obtained by comparative intergroup analysis of the microbial diversity of tobacco at the genus level, followed by dimensionality reduction analysis (Figure 5). The distribution of bacterial communities generally showed a three-stage characteristic, and the CTLs of different varieties possessed their own specificity in the Initial-Stage, W0 and F0 had low similarity, but W7 and F0 had similarity, and as fermentation proceeded, F7, F14, and W14 had high similarity, and F23, F56, F65, W23, W56, and W65 were extremely similar, and according to the PCoA analysis obtained results, the cigar fermentation was categorized into three stages: Initial-Stage, Mid-Stage, Late-Stage. The CTLs of different varieties differed in the Initial-Stage, but the microbial community structure became more and more consistent as the process progressed, but with a high degree of similarity at the end of fermentation.

As for the fungal community, the samples in each group were more dispersed compared to bacteria, without obvious stage differentiation, but still clustered into one group at the Late Stage (56d, 65d), indicating that the structure of microbial community tended to be consistent at the end of fermentation.

**Figure 6:**
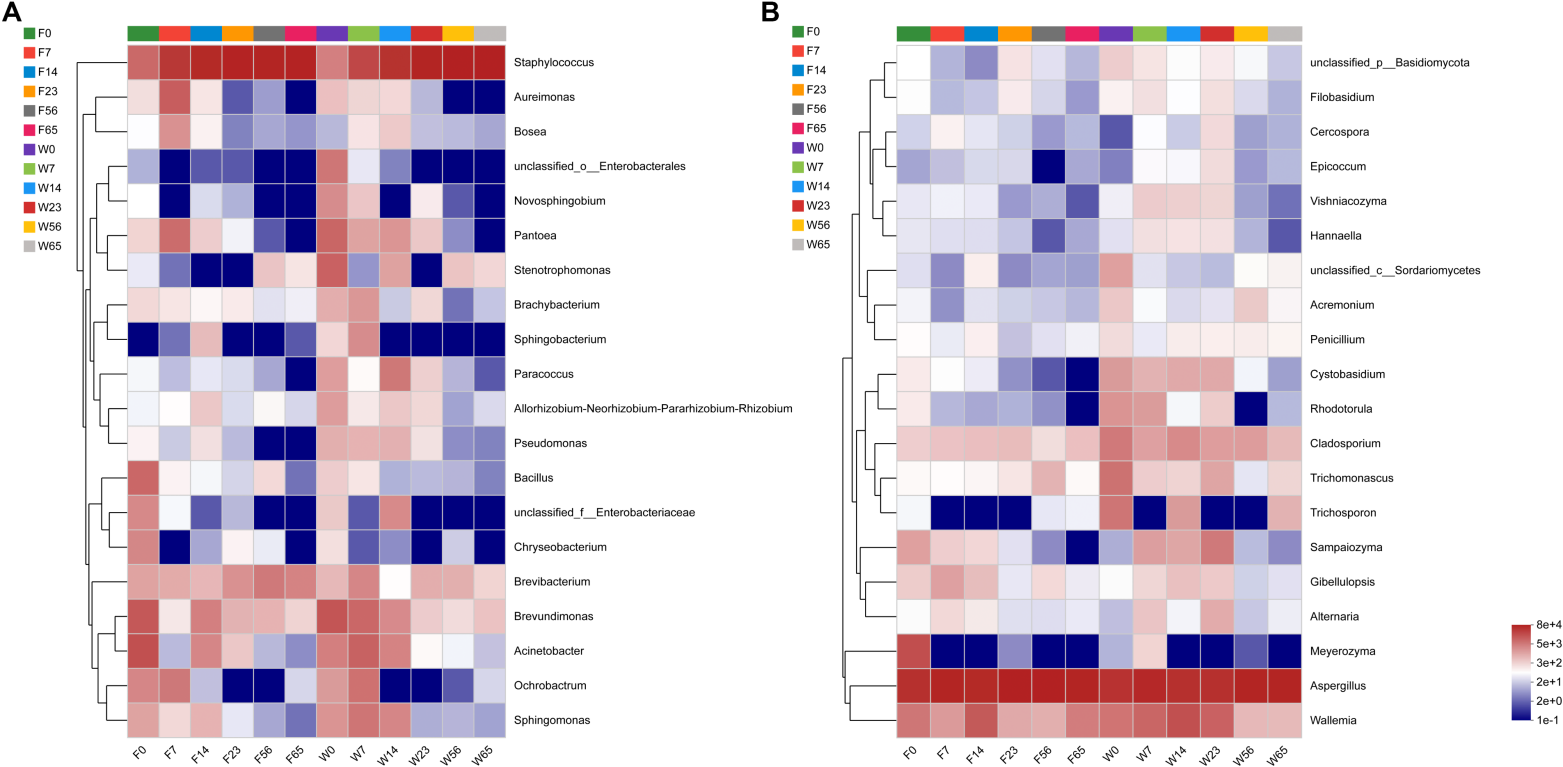
Genus-level bacterial **(A)** and fungal **(B)** community heat maps for cluster analysis.

For in-depth analysis of the bacterial and fungal community structure, the top 20 abundant genus were selected for clustering (Figure 6). This clustering facilitate a comparative assessment of the community composition across various time points. The heat map reaffirmed the convergence in the phyllosphere microbial composition of distinct CTLs species during the Late-Stage-Fermentation. During the Initial-Stage-Fermentation, specifically at 0 days, the microbial communities exhibited similar species diversity, yet there were substantial variations in the abundance of each genus, resulting in a poor overall similarity. As the fermentation progressed to the Mid-Stage, the disparities between the communities gradually diminished. In the Late-Stage of fermentation, particularly after 56 days, a marked similarity in the community composition was observed across CTLs, regardless of their fermentation duration or species. This similarity represents the culmination of the primary fermentation process for cigars. Notably, the abundance of the key microorganisms in the Wapper attained a steady state earlier than Filler, indicating a faster progression of the fermentation process in the Wapper compared to the Filler.

We conducted a comprehensive LEfSe multilevel species difference discriminant analysis on CTLs of varying species and stages (Supplementary Figure S2). The analysis aimed to discern significant differences at multiple taxonomic level (phylum, order, family, genus, and species). Furthermore, the LDA value served as a metric to quantify the extent of species’ influence on the observed differences (Supplementary Figure S3), thereby indicating a potential pivotal role played by species in the dynamic process of environmental change. With regards to the bacterial community within Wapper, characteristic species include *Staphylococcus*, *Paracoccus*, *Brachybacterium*, *Microbacterium*, *Sphingomonas*, and *Pantoea*. Among the fungal species, *Wallemia*, *Cladosporium*, *Vishniacozyma*, and *Sampaiozyma* were identified as distinctive. Conversely, in Filler, the bacterial community was characterized by the presence of *Brevundimonas*, *Oceanobacillus*, *Bosea*, *Aureimonas*, and *Bacillus*, with *Aspergillus* being the sole distinctive fungal species. Importantly, both *Aspergillus*, the most abundant fungal genus, and the bacterial genus *Staphylococcus* exhibited notable roles in varying CTLs genotypes. However, their influence was particularly pronounced in Filler.

**Figure 7:**
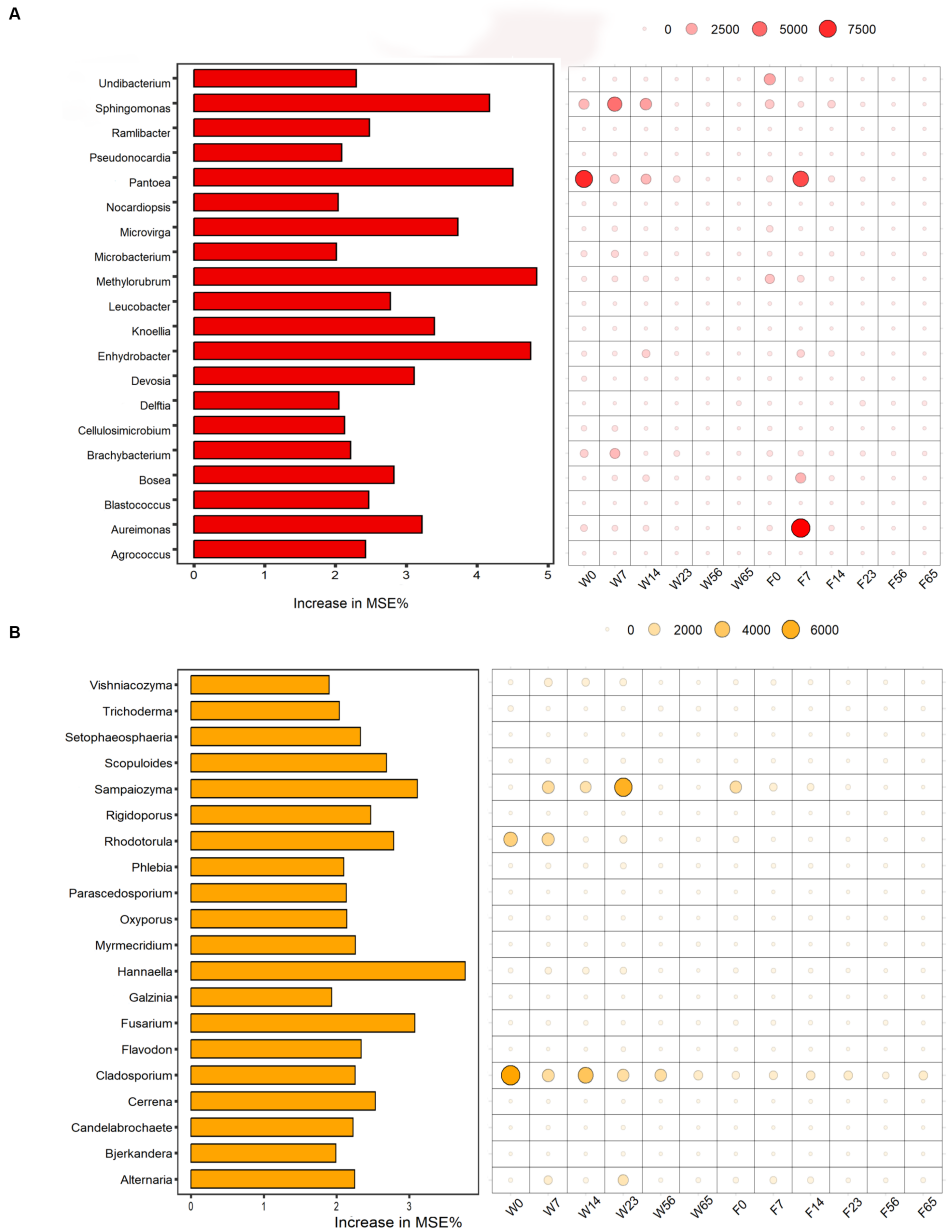
Random forest algorithm to calculate biomarkers for bacterial **(A)** and fungal **(B)** community in cigar fermentation processes.

Using the LEfSe analysis as a basis, a random forest algorithm was employed to precisely identify signature microorganisms and discover differential biomarkers at each stage (Figure 7). The Mean Decrease Accuracy metric quantifies the extent of reduction in the random forest’s predictive accuracy when a variable’s value is randomly perturbed, with higher values indicating greater significance of that variable. The left-sided bar graph depicts the significance of the biomarkers at the genus level, while the bubble graph on the right portrays the importance of each differential bacterial genus at specific stages. Given the high significance of the *Staphylococcus* genus across all stages, in comparison to other dominant bacterial genera, we excluded its effect. In the pre-fermentation period, *Pantoea* emerged as a prominent signature microorganism, followed by *Sphingomonas*. Notably, Filler exhibited unique signature microorganisms in the Initial-Stage, particularly *Aureimonas*, a genus that was not significantly represented in Wapper. Furthermore, we observed that the signature fungi were nearly absent in Filler compared to Wapper, where *Rhodotorula* was identified as the signature fungi in the Initial-Stage, *Sampaiozyma* during the Mid-Stage, and *Cladosporium* was prominent in both the pre- and mid-fermentation phases.

### 3.5 Chemical composition of fermentation

**Figure 8:**
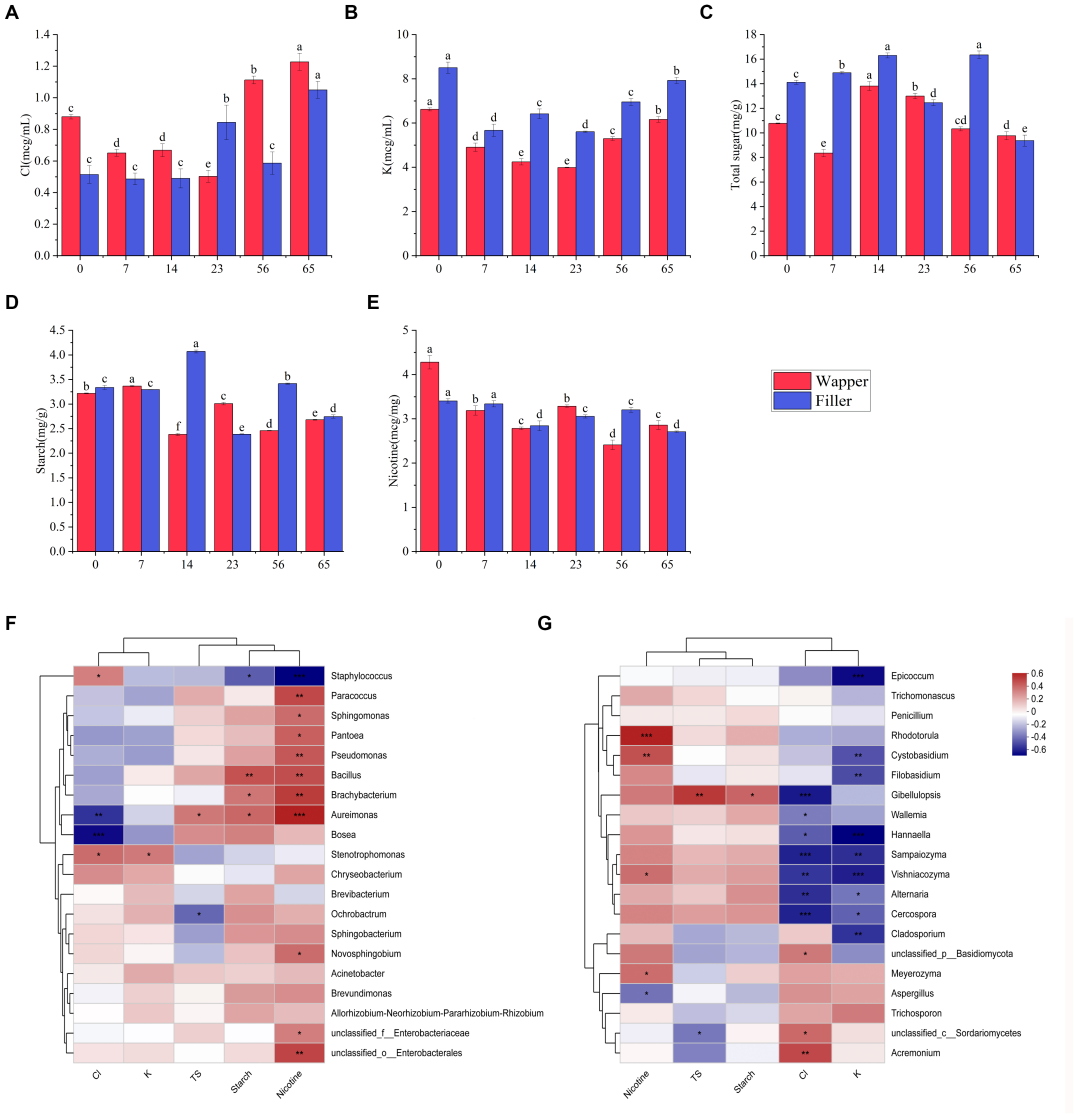
Changes in chemical content during fermentation: **(A)** chlorine, **(B)** potassium, **(C)** starch, **(D)** total sugars, and **(E)** nicotine; correlation of top 20 dominant bacterial genera **(F)** and fungal genera **(G)** with chlorine, potassium, starch, total sugars, and nicotine content. 0, 7, 14, 23, 56, and 65 is the duration of fermentation.

We take an analysis was conducted to determine the concentrations of chloride, potassium, total sugar (TS), starch, and nicotine among different fermentation stages. The outcomes revealed a distinctive pattern in the chloride, initially exhibiting a decline followed by an increase, culminating in a significant augmentation in the end of process, this pattern was consistently observed across diverse varieties (Figure 8A). Analogous to chloride content, potassium content also exhibited a similar trend of initial decrement and subsequent increment, with the notable difference that chloride concentrations remained consistently higher throughout the process, while potassium concentrations were lower in Wapper compared to Filler (Figure 8B). Total sugar content, a crucial indicator for assessing the quality of cigar tobacco, displayed an intermediate rise in both samples, yet underwent a sharp decline towards the Late-Stage, ultimately significant failing below the initial levels. Notably, despite a significant disparity in the initial total sugar content between the two samples, their concentrations converged towards the end of the process, as depicted in Figure 8C. Starch exhibited a similar trend to total sugar, displaying sharp fluctuations in the Mid-Stage, but stabilized in the Late-Stages (Figure 8D). Conversely, nicotine content gradually decreased throughout the process, with a marked decrement at the culmination of fermentation. Notably, Wapper possessed a higher nicotine content compared to Filler in the end (Figure 8E).

The analysis revealed a discernible pattern of correlation between the dominant bacterial genera and their chemical composition (Figures 8F,G). Specifically, *Staphylococcus* exhibited a notable positive correlation with the chlorine content and a negative correlation with nicotine, total sugar, starch, and potassium. Among these parameters, the association with nicotine was particularly significant, followed closely by starch. Conversely, *Bacillus* displayed a significant positive correlation with starch and nicotine, while exhibiting a negative correlation with chlorine. *Aureomonas* showed a significant positive correlation with total sugar, starch, nicotine, and a significant negative correlation with chlorine content. *Bosea* exhibited a significant negative correlation with chlorine content. *Ochrobacterium*, on the other hand, displayed a significant negative correlation with total sugar. Furthermore, the genera *Paracoccus*, *Sphingomonas*, *Pantoea*, and *Pseudomonas*, as well as *Brachybacterium*, all exhibited significant positive associations with nicotine. In contrast to the intimate association observed between bacteria and organic compounds, fungi exhibit a stronger correlation with chlorine and potassium. Among the top 20 fungal genera in abundance, 14 genera exhibited a significant correlation with either chlorine or potassium, predominantly displaying a negative correlation. However, exceptions were noted in *Acremonium* and *Branchiostoma*, which showed a significant positive correlation with chloride, these fungi maintained a relatively low abundance throughout the process. Additionally, fungi exhibited a notable correlation with nicotine content, with the exception of *Aspergillus*, which displayed a significant negative correlation. Conversely, *Rhodotorula*, *Cystobasidium*, and *Vishniacozyma* all exhibited significant positive correlations with nicotine.

### 3.6 Metabolites during fermentation

**Figure 9:**
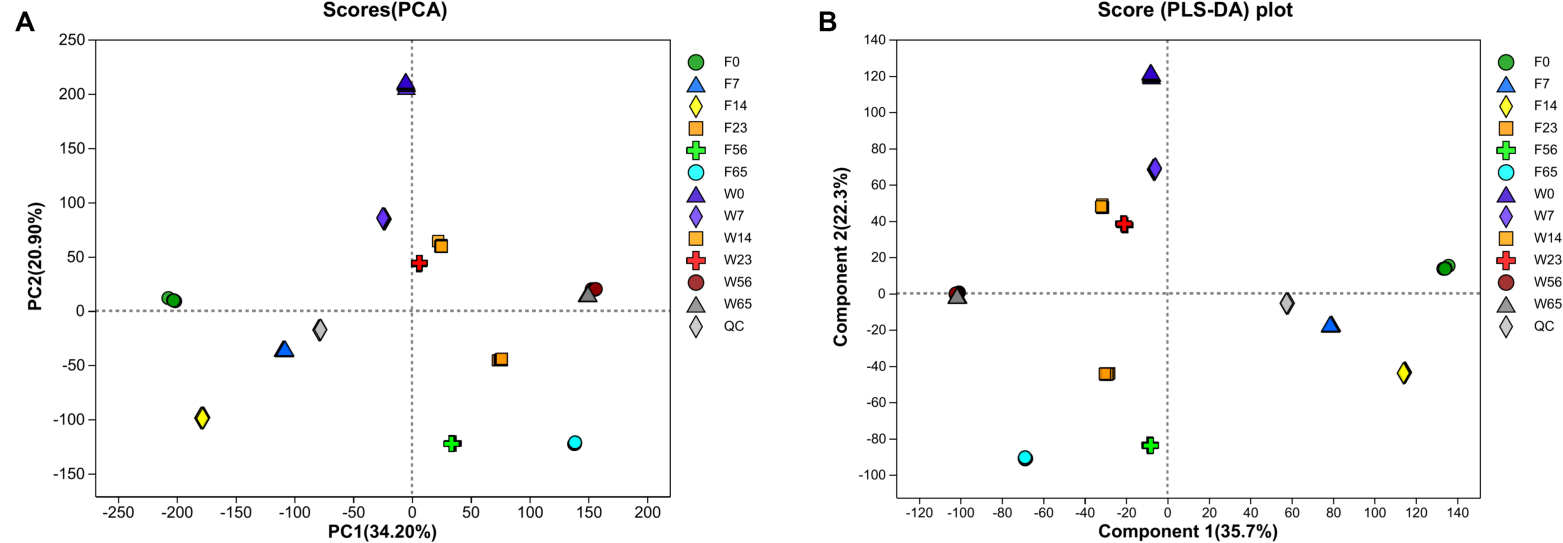
Principal component analysis **(A)** and partial least squares discriminant analysis **(B)** to map whole process metabolism.

In the course of the fermentation process, metabolomic analysis identified a total of 1,219 metabolites, encompassing 589 terpenoids, 167 steroids, 111 phenolic acids, 82 organic acids, 70 indoles, 63 flavonoids, 56 alkaloids, 38 coumarins, 18 lignans, 15 quinones, 8 stilbenes, 2 ellagitannins, and 2 tannins. The application of Principal Component Analysis (PCA) revealed a distinct segregation of metabolites among the various samples within each group, indicating significant variations in the metabolic profiles of CTLs of diverse varieties at different stages (Figure 9). Notably, the Wapper samples attained a metabolic steady state at an earlier stage, with the metabolite distributions converging at 56 and 65 days. This observation was further corroborated by Partial Least Squares Discriminant Analysis (PLS-DA).

**Figure 10:**
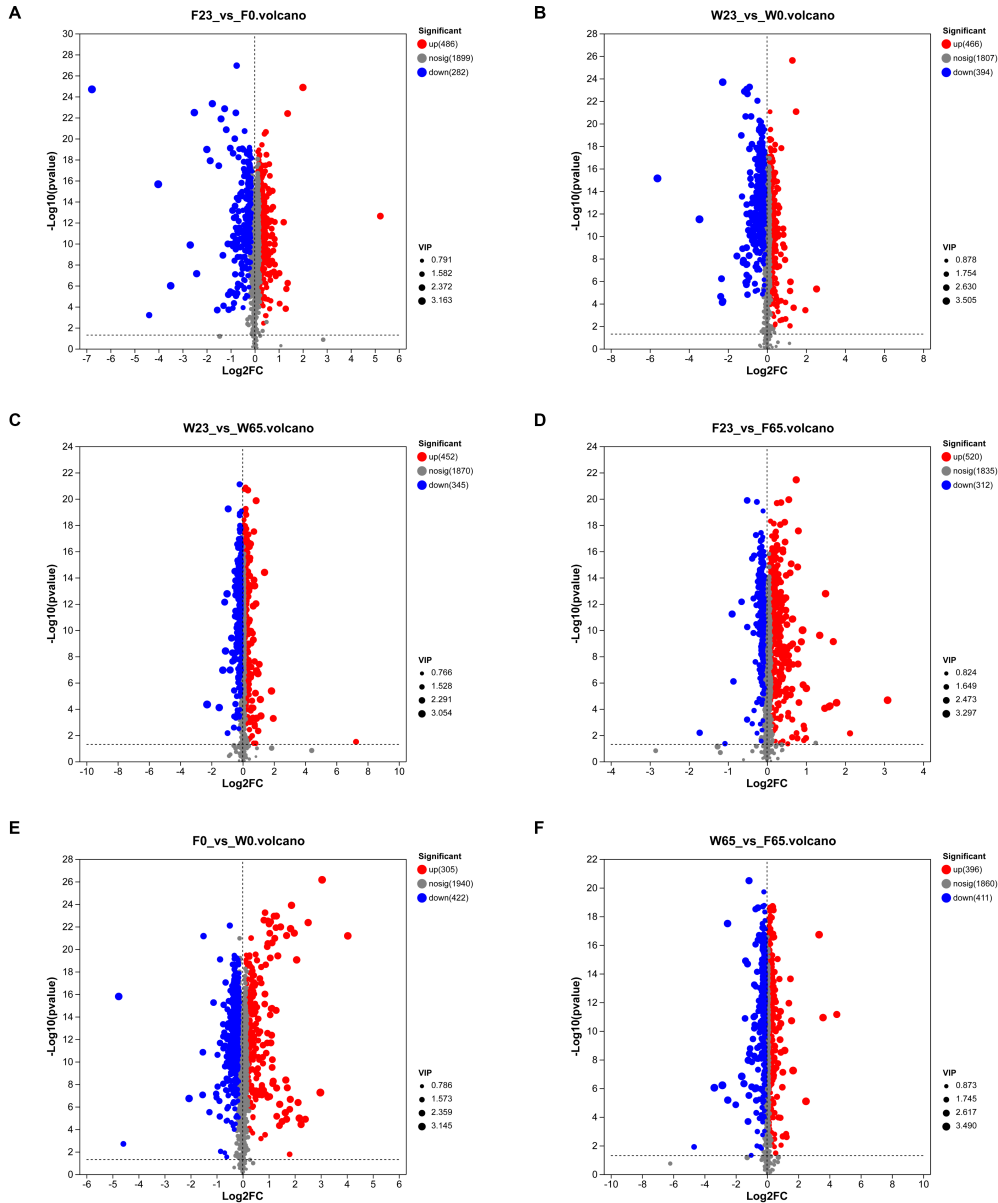
Differential metabolite volcano plots of controls between groups **(A)** F23 vs. F0, **(B)** W23 vs. W0, **(C)** W23 vs. W65, **(D)** F23 vs. F65, **(E)** F0 vs. W0, and **(F)** W65 vs. F65; each point in the plots represents a specific metabolite, and the magnitude of the point indicates the VIP value. Red points indicate significantly up-regulated metabolites, blue points indicate significantly down-regulated metabolites, and gray points are non-significantly different metabolites.

A rigorous approach involving both univariate and multivariate statistical analyses was implemented to screen for differential metabolites across the two distinct biomes. A representative time node from various stages of the same cultivar was selected for pairwise comparative analysis. Similarly, this comparative analysis was also conducted for the metabolite profiles of Wapper and Filler at 0 and 65 days, representing the pre- and post-fermentation states (Figure 10). The analysis revealed 768 differential metabolites in F23 vs. F0 (up = 486, down = 282), and 832 differential metabolites in F23 vs. F65 (up = 520, down = 312). 860 differential metabolites were observed in W23 vs. W0 (up = 466, down = 394), along with 797 differential metabolites (452 upregulated, 345 downregulated). 727 differential metabolites in F0 vs. W0 (up = 305, down = 422), and 807 differential metabolites in W65 vs. F65 (up = 396 down = 411). The metabolic activity on the cigar leaf surface peaked during the mid-fermentation stage, highlighting its crucial role in the initial cigar fermentation process. The comparison of differential metabolite indices between the two cultivars at 0d and 65d suggested that Wapper exhibited a more robust metabolism at Initial-Stage but trailed behind Filler towards the Late-Stage. This finding aligns with the relatively faster completion of the fermentation in Wapper, consistent with the microbial community heatmap and PCA/PLS-DA metabolite clustering results.

**Figure 11:**
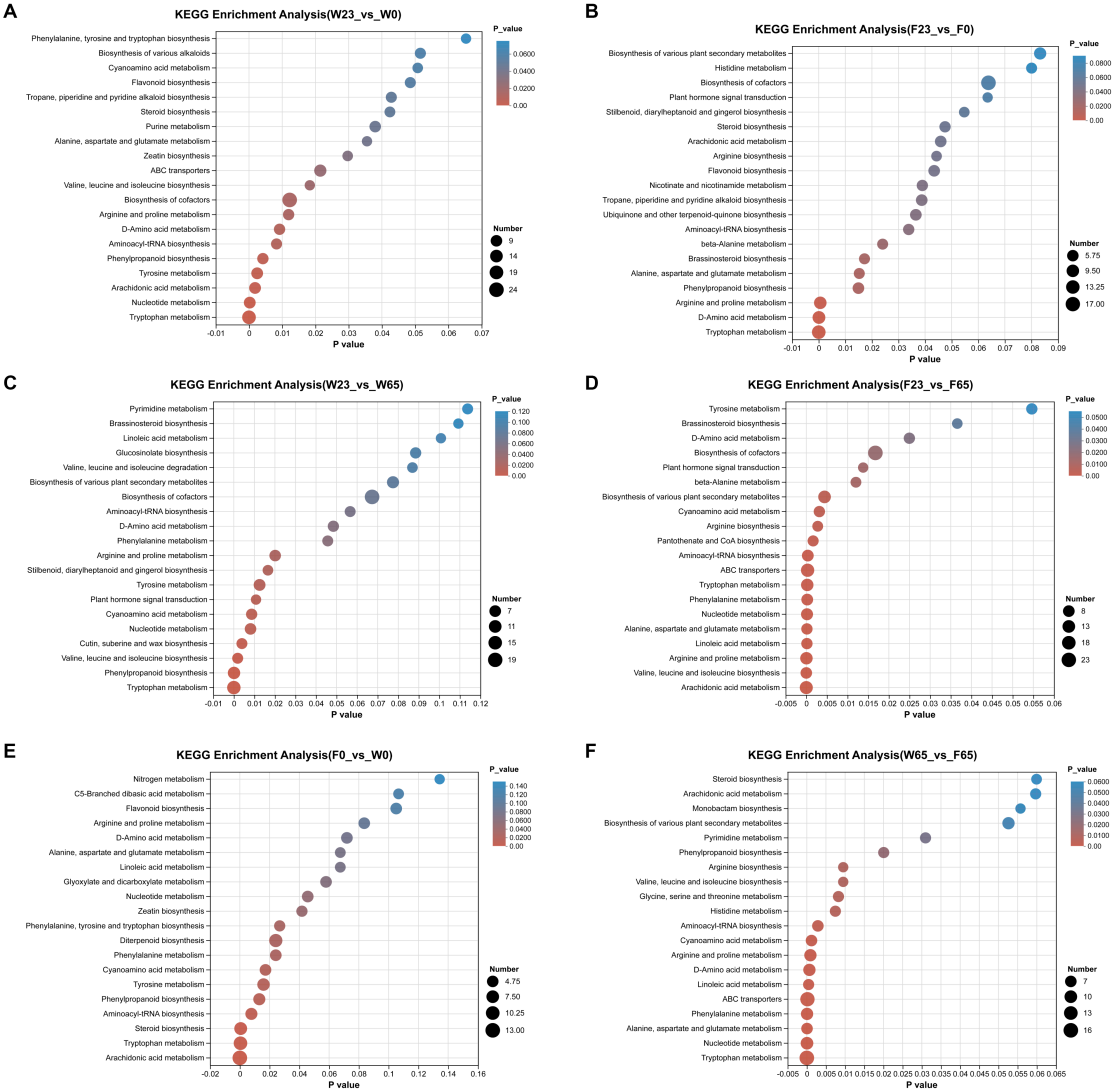
KEGG metabolic pathway enrichment between groups **(A)** F23 vs. F0, **(B)** W23 vs. W0, **(C)** F23 vs. F65, **(D)** W23 vs. W65, **(E)** F0 vs. W0, and **(F)** W65 vs. F65; the horizontal coordinates are the *p*-value of the significance of the enrichment, and the smaller *p*-value is statistically significant, and a *p*-value of less than 0.05 is generally considered to be a function of a significantly enriched term; vertical coordinate is KEGG pathway. The size of the bubbles in the graph represents how much of the pathway is enriched to the metabolic set of compounds.

Utilizing the KEGG database, we were able to perform enrichment analysis on the identified differential metabolites, leading to the identification of significant metabolic pathways during the process (Figure 11). In the comparison between different stages, the metabolic pathways that were significantly enriched in the W23 vs. W0 are “Biosynthesis of cofactors,” “Tryptophan metabolism,” “Nucleotide metabolism,” “Arachidonic acid metabolism,” “Tyrosine metabolism.” The metabolic pathways that were significantly enriched in the W23 vs. W65 are “Tryptophan metabolism,” “Phenylpropanoid biosynthesis,” “Valine, leucine and isoleucine biosynthesis,” “Cutin, suberine and wax biosynthesis,” “Nucleotide metabolism.” The metabolic pathways that were significantly enriched in the F23 vs. F0 are “Arginine and proline metabolism,” “D-Amino acid metabolism,” “Tryptophan metabolism.” The metabolic pathways that were significantly enriched in the F23 vs. F65 are “Arachidonic acid metabolism,” “Valine, leucine and isoleucine biosynthesis,” “Arginine and proline metabolism,” “Linoleic acid metabolism,” “Alanine, aspartate and glutamate metabolism,” “Nucleotide metabolism,” “Phenylalanine metabolism,” “Tryptophan metabolism,” “ABC transporters,” “Aminoacyl-tRNA biosynthesis.”

When comparing between different varieties, the metabolic pathways that were significantly enriched in the F0 vs. W0 are “Arachidonic acid metabolism,” “Tryptophan metabolism,” “Diterpenoid biosynthesis,” “Steroid biosynthesis,” “Aminoacyl-tRNA biosynthesis.” The metabolic pathways that were significantly enriched in the F0 vs. W0 are “Tryptophan metabolism,” “Nucleotide metabolism,” “Alanine, aspartate and glutamate metabolism,” “Phemtlalanine metabolism,” “ABC transporters,” “Linoleic acid metabolism,” “Phenylalanine metabolism,” “Arginine and proline metabolism,” “cyanoamino acid metabolish.”

As depicted in the Wayne diagram (Supplementary Figure S4), a mere 35 metabolites were found to be shared among the comparison groups of stages and genotypes, indicating a high degree of specificity in the majority of differential metabolites, necessitating further analysis. To elucidate these metabolites, the Top-20 metabolites between groups were selected for clustering analysis (Supplementary Figure S5). In the W23 vs. W0 comparison, 10 up-regulated metabolites included 4 amino acids, 2 others, 2 vitamins, 1 terpene, and 1 indole, while 10 down-regulated metabolites comprised 3 others, 3 terpenes, 2 lipids, 1 carbohydrate, and 1 amino acid. In W23 vs. W65, 11 up-regulated metabolites encompassed 4 lipids, 1 indole, 1 carbohydrate, 1 amino acid, 1 steroid, 1 nucleotide, 1 organic acid, 1 others, and 9 down-regulated metabolites comprised 6 terpenes, 1 carbohydrate, 1 lipid, and 1 others. In F23 vs. F0, 8 up-regulated metabolites consisted of 4 lipids, 2 carbohydrates, and 2 others, while 12 down-regulated metabolites comprised 4 others, 4 terpenes, 2 lipids, 1 steroid, and 1 carbohydrate. Finally, in F23 vs. F65, 10 up-regulated metabolites comprised 7 lipids, 2 steroids, and 1 others, and 10 down-regulated metabolites consisted of 3 lipids, 3 others, 2 terpenoids, 1 amino acid, and 1 carbohydrate.

Between genotypes, W0 vs. F0 revealed 10 up-regulated metabolites comprising 3 amino acids, 2 vitamins, 2 others, 2 organic acid, and 1 indole, while 10 down-regulated metabolites encompassed 4 lipids, 4 terpenes, and 2 others. In W65 vs. F65, 12 up-regulated metabolites were identified, including 5 lipids, 3 amino acids, 1 organic acid, 1 nucleotide, 1 terpene, and 1 others. The 8 down-regulated metabolites comprised 6 lipids, 1 steroid, and 1 others.

### 3.7 Correlation of metabolites with microbiota

**Figure 12:**
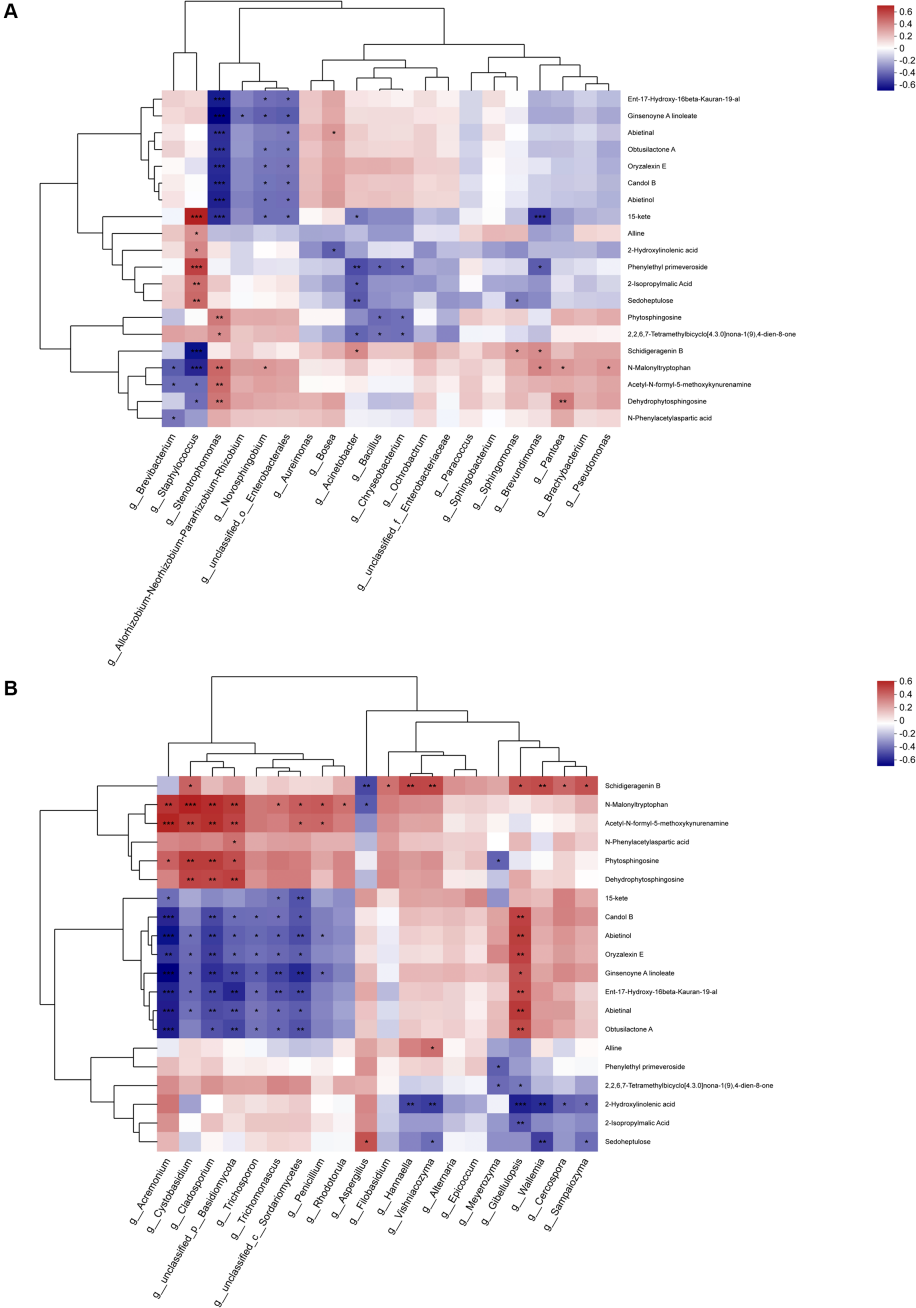
Association analysis of top 20 metabolites during fermentation with **(A)** dominant bacterial genera and **(B)** dominant fungal genera.

The top 20 metabolites were rigorously selected and subjected to an in-depth analysis, taking into account their associations with the predominant bacterial and fungal genera (Figure 12). The metabolites associated with the dominant bacterial genera can be categorized into three cluster across the entire metabolic profile. Cluster 1 was primarily composed of terpenes, cluster 2 encompassed lipids, indoles, and carbohydrates, while cluster 3 was primarily dominated by amino acids. Notably, the interplay between the substances in cluster 1 and the dominant genera exhibited a complex pattern, with the numbers of bacteria displaying positive and negative correlations are largely equivalent, yet the majority of significant correlations are predominantly negative. Such as *Stenotrophomonas* and *Novosphingobium*. In cluster 2, while most substances displayed negative correlations with the dominant genera, the most abundant genus, *Staphylococcus*, exhibited a significant positive correlation. Additionally, *Brevibacterium* also displayed a positive correlation with most substances. In cluster 3, most substances displayed positive correlations with the dominant genera, particularly with *Stenotrophomonas* and *Pantoea*, yet exhibited significant negative correlations with *Staphylococcus* and *Brevibacterium*.

In analyzing the fungal genera, a similar categorization of the top 20 metabolites into three cluster was observed. Cluster 1 primarily encompassed amino acids, cluster 2 primarily comprised terpenes and lipids, while cluster 3 primarily contained indoles, carbohydrates, and lipids. The metabolites within cluster 1 displayed positive correlations with most dominant fungal genera, particularly exhibiting significant positive correlations with *Acremonium*, *Cystobasidium* and *Cladosporium*, a few metabolites exhibited significant negative correlations with *Aspergillus*.

The 4 fungal species exhibiting significant positive correlations with metabolites in cluster 1 displayed contrasting behaviors in cluster 2, demonstrating marked negative correlations with all metabolites, analogous to the patterns observed in *Trichosporon* and *Trichomonascus*. In contrast, all genera apart from *Trichosporon* displayed positive correlations with metabolites, particularly with *Gibellulopsis*. However, in cluster 3, the correlations between metabolites and the dominant genera were comparatively minor, with only *Meyerozyma*, *Gibellulopsis*, and *Wallemia* exhibiting significant negative correlations with most metabolites. Notably, the important genus *Aspergillus* displayed positive, albeit non-significant, correlations with cluster 3. Furthermore, the association between metabolites in cluster 3 and the dominant genera was relatively insignificant, with only *Meyerozyma*, *Gibellulopsis*, and *Wallemia* exhibiting significant negative associations with most metabolites, while the significant genus *Aspergillus* exhibited a positive association with cluster 3, though the correlation was not statistically significant.

### 3.8 Overview of association networks between important microorganisms

**Figure 13:**
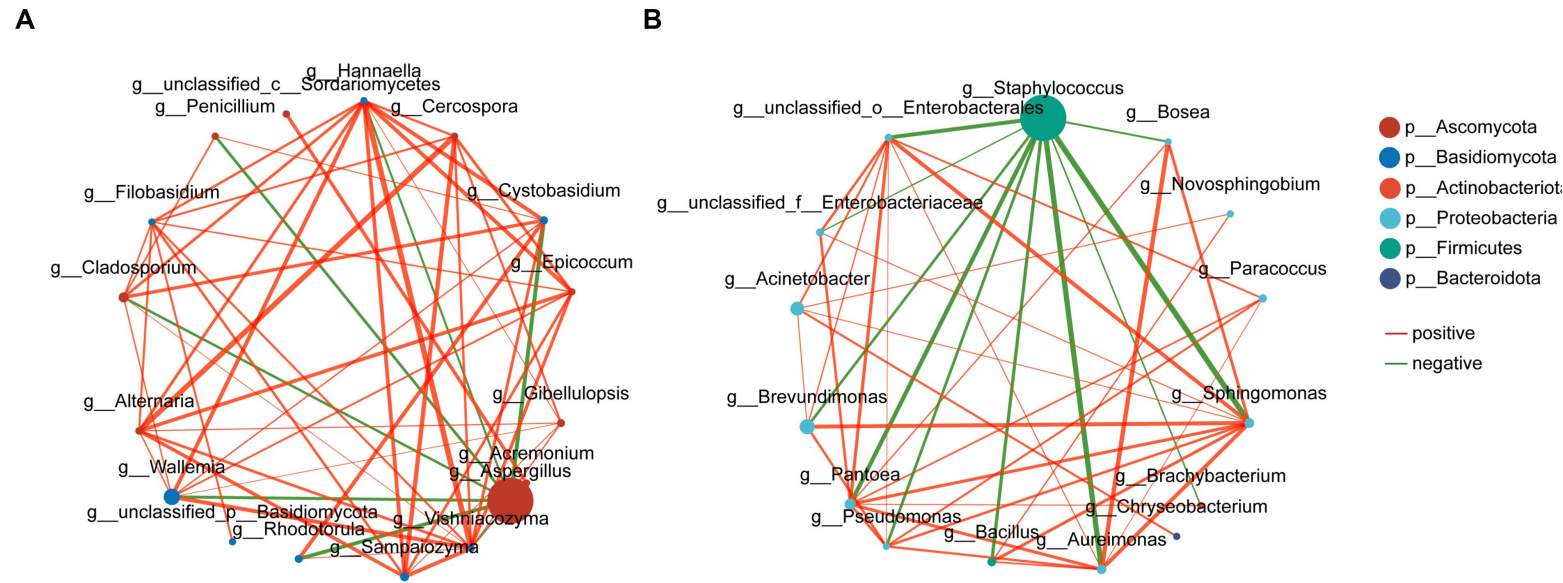
Network diagram of correlations between fungal **(A)** and bacterial **(B)** microbial communities. Red curves indicate positive correlations and green curves indicate negative correlations.

Network analysis was used to show the distribution of species, and the 15 most abundant bacterial and fungal genera throughout the phase were selected for correlation network mapping. Among the bacterial communities, *Staphylococcus* was the antagonistic centre of the bacteria and was negatively correlated with most of the other dominant genera, while *Sphingomonas* was the synergistic centre of the bacteria and was significantly positively correlated with the rest of the dominant genera except *Staphylococcus*. In the fungal community, *Aspergillus* showed the characteristics of an antagonistic centre, whereas *Wallemia* and *Hannaella*, although accounting for a smaller percentage of the abundance, still acted as synergistic centres for the fungi (Figure 13).

### 3.9 Important metabolic pathways

**Figure 14:**
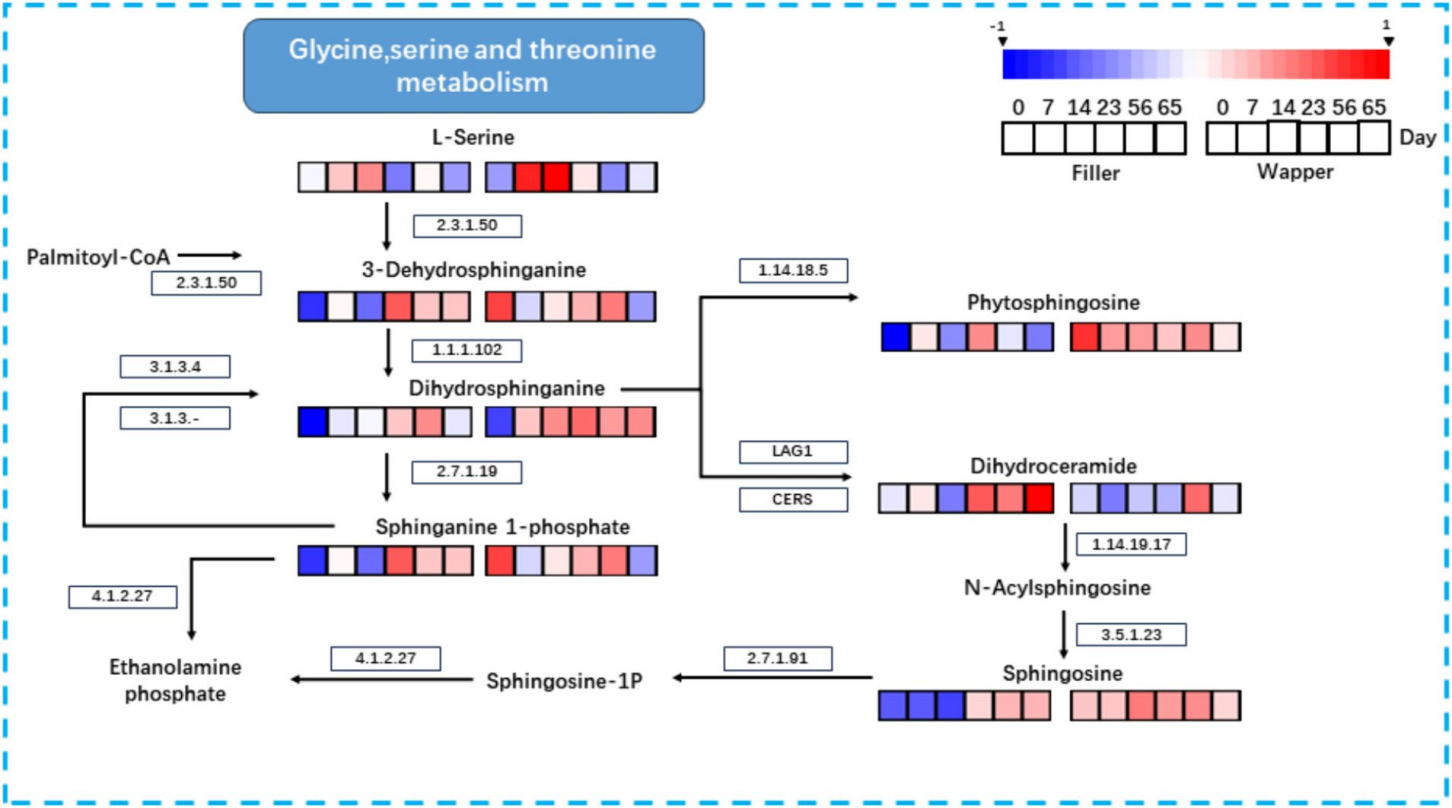
Metabolic mapping of important metabolites during fermentation of CTLs: sphingolipids from glycine, serine, and threonine metabolism.

To elucidate the intricate relationship between microorganisms and their metabolites, we harnessed the Kyoto Encyclopedia of Genes and Genomes (KEGG) database to delineate the pertinent metabolic networks of significant substances during the initial fermentation phase of CTLs (Figure 14). The metabolic pathways were comprehensively mapped by integrating the KEGG database with the outcomes of non-targeted metabolite profiling.

Specifically, we focused on the key metabolite phytosphingosine and traced its associated metabolic pathway. The biosynthetic process commences with the condensation of serine and palmitoyl coenzyme A, catalyzed by EC:2.3.1.50 (serine palmitoyltransferase), to yield 3-Dehydrosphinganine. Subsequently, EC:1.1.1.102 reduces 3-Dehydrosphinganine to Dihydrosphinganine. The metabolic trajectory of Dihydrosphinganine diverges into three distinct pathways: one leading to Phytosphingosin via EC:1.14.18.5 (sphinganine C4-monooxygenase); another towards Phytosphingosin through the condensation of sphinganine 1-phosphate by EC:2.7.1.91 (sphingosine kinase) and its interconversion with phosphatidate phosphatase (EC:3.1.3.4); and finally, the combination of LAG1 and CERS generates Dihydroceramide, the precursor of the two aforementioned metabolites. Dihydroceramide is further metabolized by EC:1.14.19.17 (sphingolipid 4-desaturase) to form N-Acylsphingosine, which is subsequently converted to Ceramide by EC:3.5.1.23 (alkaline ceramidase). Notably, within this sphingolipid metabolic cascade, no further metabolites were identified until the termination of fermentation, wherein the enrichment of metabolites primarily occurred in Phytosphingosin, Sphingosine, and Sphinganine 1-phosphate (S1P). S1P, a crucial signaling molecule, possesses the capability to modulate both pathophysiological and physiological processes, thus rendering it of immense importance in the treatment of various diseases and malignancies (Winkler et al., 2017; Zheng et al., 2019).

## 4 Discussion

The utilization of fermentation processes has been demonstrated to substantially enhance the quality of CTLs (Zhang et al., 2024). In the current investigation, a comprehensive analysis was conducted on the microbial community composition of CTLs during fermentation, along with the alterations in tobacco chemical constituents and metabolites. This analysis serves as a theoretical foundation for elucidating the microbial mechanisms underlying primary fermentation and facilitates the development of novel fermentation microbial preparations.

The dynamic changes in the chemical composition and metabolic substances of CTLs were achieved through the synergistic action of diverse microorganisms (Liu et al., 2024). During the entire fermentation, five primary bacterial genera were identified: *Staphylococcus*, *Brevundimonas*, *Acinetobacter*, *Brevibacterium*, and *Pantoea*. Additionally, five fungal genera were detected, including *Aspergillus*, *Meyerozyma*, *Sampaiozyma*, *Cladosporium*, and *Trichomonascus*. In previous research, the dominant flora were frequently recognized as playing a pivotal role in the fermentation (Liu et al., 2023). *Staphylococcus* exhibited remarkable adaptability, maintaining a higher abundance during fermentation (Mazharul Islam et al., 2020). Notably, along with *Aspergillus*, their abundance progressively increased from the Initial-Stages, ultimately occupying a dominant position throughout the entire process. The secretion of α-Amylase and amylase by these two genera was the primary reason behind the alterations in starch and total sugar content (Lakshmi et al., 2013). Furthermore, through correlation analysis with chemical content, it is postulated that the enzymatic activities of these genera contribute to the degradation of nicotine, a process that generates pyrrole, pyridine, and pyrazine compounds, which significantly influence the aroma of tobacco leaves (Yang et al., 2021). Elevated chlorine content in tobacco has been documented to significantly compromise its combustibility (Su et al., 2024). Less abundance genera such as *Bacillus*, *Aureimonas*, and *Alternaria* exhibit a negative correlation with the chlorine content, this observation stands in contrast to the behavior exhibited by *Staphylococcus*, underscores the importance of these genera in improving the overall tobacco quality. This finding suggests that during the fermentation process, the abundance of dominant genera like *Staphylococcus* should be meticulously managed to prevent undue disruption to the microbial diversity, thereby optimizing the quality enhancement of tobacco.

The intricate interplay between microorganisms, their metabolites, and the complex interactions among microorganisms is a pivotal determinant of the quality of CTLs (Singh et al., 2017) A non-targeted metabolomics analysis revealed that metabolites with a significant percentage in the fermentation process encompass terpenoids, steroids, phenolic acids, organic acids, and indoles, whereas flavonoids, alkaloids, coumarins, and lignans constitute a smaller proportion. Notably, terpenoids are a crucial source of aroma in CTLs (Gao et al., 2023). For instance, monoterpenes, the primary components of plant aroma, can be extracted for use in flavoring and fragrances (Yin et al., 2017). Cembratrienes, significant terpenes synthesized during tobacco growth, yield solanone as a degradation product, which is a key aroma-inducing component in tobacco (Sui et al., 2018; Popova et al., 2020). In contrast to previous studies, our findings indicate a higher abundance of indoles among the metabolites, with Alline emerging as a leading metabolite among the top 20. Indoles are a class of signaling molecules and regulators produced by bacteria through the degradation of amino acids (L-tryptophan) by tryptophanase (TnaA) (Lee et al., 2015). These indole derivatives, such as indole alkaloids, further regulate microbial physiological processes and indire CTLs influence the microbial community structure, for instance, by enhancing the viability of the fermentation-dominant bacterium *Pantoea* (Zheng et al., 2017). Moreover, they improve tobacco’s resistance to mold occurrence and attack (Ma et al., 2024). *Staphylococcus* and *Aspergillus* are the main contributors of peptidases during fermentation, participating in the hydrolysis of proteins to produce peptides and amino acids, which serve as precursors of flavor substances (Zhao et al., 2019; Jia et al., 2021), and *Staphylococcus* is also an important participant in the metabolism of amino acid catabolism that can significantly accelerate the development of aroma (Bruna et al., 2000; Aro Aro et al., 2010). *Acinetobacter* is able to increase the ability of microbial communities to break down macromolecules while producing aldehydes and ketones associated with aroma (Zheng et al., 2022). *Brevundimonas* is able to catabolise starch in a combined action with strains such as *Bacillus* under suitable temperature environment (Ma and Su, 2019). *Bacillus* are able to break down substances such as glucose to synthesize glutamate, which increases the flavor of the product (Nampoothiri and Pandey, 1996). *Cladosporium* is an important filamentous fungus that produces amylase, protease and cellulase enzymes, which are active in the decomposition process of plants (Abe et al., 2015).

Drawing insights from the assessment of α-diversity and β-diversity of microorganisms, as well as the observed trends in microbial structural variations, the fermentation process of CTLs was systematically categorized into three distinct phases: the Initial-Stage, Mid-Stage, and Late-Stage. Within each of these stages, a representative time point was deliberately selected to facilitate comparative analyses, thereby providing a comprehensive understanding of the microbiological dynamics throughout the fermentation process. In the comparative analyses of F23 vs. F0 and W23 vs. W0, the predominant enriched metabolic pathways were associated with general “metabolic pathways.” However, in F23 vs. F65 and W23 vs. W65, these pathways were augmented by a diverse array of “biosynthetic pathways,” such as “Valine, leucine and isoleucine biosynthesis,” “Cutin, suberine and wax biosynthesis,” “Nucleotide metabolism.” This disparity might stem from the observation that in the initial-to-intermediate phase, chemical transformations within CTLs are primarily directed towards substance decomposition, whereas in the intermediate-to-late phase, substance synthesis becomes the primary focus. Upon examining the differential metabolites within the top 20 between groups, it was noted that in the 23d versus 0d comparison, lipids and amino acids constituted the primary up-regulated metabolites. Similarly, in the 23d versus 65d comparison, lipids remained the dominant up-regulated metabolites, and with a higher percentage. This observed phenomenon could potentially be attributed to the proliferation of lipolytic *Staphylococcus* during the intermediate stages of fermentation (Jeong et al., 2014). These bacteria actively break down macromolecular lipids into their corresponding esters and proteins into branched-chain amino acids (Zhao et al., 2022). Consequently, in a correlation analysis conducted on the top 20 metabolites with the dominant bacterial genera throughout the entire fermentation process, *Staphylococcus* were found to be associated with a majority of the lipids and amino acids in the comparative group, specifically exhibited a significant positive correlation with the majority of lipids. It is noteworthy that the primary aroma sources in processed foods and products are ketones derived from amino acid degradation and aldehydes resulting from lipid oxidation (Korecka et al., 2016) this aligns with the upregulation of metabolites such as amino acids and lipids during the Mid-Stage, indicating that the Mid-Stage is crucial in regulating the aroma quality of CTLs.

Despite their relatively low abundance during fermentation, *Stenotrophomonas* species have been established as closely associated with various metabolites. Prior investigations have revealed their significant potential to enhance the content of volatile aroma components (Zhang et al., 2024). In the present study, a highly significant negative correlation was observed between *Stenotrophomonas* and the majority of terpenes, presumably due to the degradation of terpenes by *Stenotrophomonas* into ketoaldehydes, which are aroma-contributing compounds (Molina et al., 2013). Furthermore, *Stenotrophomonas* displayed a remarkable positive correlation with Acetyl-N-formyl-5-methoxykynurenamine (AFMK), a melatonin metabolite that elevates antioxidant levels in organisms. AFMK is widely acknowledged for its superior antioxidant capacity compared to carotenoids and vitamin C (Galano et al., 2013; Reina and Martínez, 2018).

Notably, sphingolipids, which constitute a significant portion of the top 20 metabolites, also exhibited a significant positive correlation with *Stenotrophomonas*. As bioactive molecules, sphingolipids play a crucial role in regulating intracellular processes, including cell proliferation and differentiation, developmental apoptosis, and immunological effects (Aguilera-Romero et al., 2014). To gain a deeper understanding of their changes during fermentation, we delved into their metabolic network using the KEGG database (Figure 10). Our research revealed that key sphingolipid metabolites, such as Phytosphingosin, Sphingosine, and Sphinganine 1-Phosphate, are closely linked to the occurrence and management of smoking-induced lung-related diseases. To a certain extent, these metabolites can mitigate the damage to the lungs caused by harmful substances generated by smoking (Chen et al., 2014; Li et al., 2020). However, during the initial and intermediate stages of fermentation (0–14 days), they under investigation were notably low, a significant enrichment was observed during the mid-to-late fermentation stages (23–65 days), which can potentially be attributed to the progressively active microbial metabolic activities commencing from the Mid-Stage.

In addition to their individual roles in fermentation, interactions between microorganisms significantly influence the whole process (Smid and Lacroix, 2013). *Sphingomonas*, *Pantoea*, *Aureimonas*, *Cladosporium*, *Sampaiozyma* are all biomarker (rf) in fermentation. They are widely present in Initial-Stage and Mid-Stage. These microorganisms showed synergistic relationships with most of the microorganisms in the mutualistic network, and their important roles in the fermentation process have been confirmed in previous studies.

It is noteworthy that our research suggest that Wapper reaches steady state earlier in the fermentation, progresses more rapidly than Filler, and possesses more fungal biomarker, which represents a possible need to tailor treatments for different genotypes of plant leaves to ensure that their quality is maintained at an optimal level.

## 5 Conclusion

In this study, through the utilization of high-throughput sequencing and non-targeted metabolomics analysis, it was determined that biomarkers are predominantly present during the Initial-Stage, whereas the microbial activity peaks during Mid-Stage (14–23d). *Staphylococcus* have a strong correlation with lipids and amino acids, dominating the microbial community, it may has effects on fermentation, we should adopt appropriate measures to constrain the proliferation of *Staphylococcus*. Additionally, *Stenotrophomonas* despite their lower abundance, play a significant role and exhibit a strong association with terpenoid metabolites. Sphingolipid metabolites are actively involved during fermentation, contribute significantly to enhancing the quality of CTLs. This investigation delved into the intricate relationship between metabolites and microorganisms, laying a theoretical foundation for further refining the fermentation process of plant leaves and advancing technologies aimed at elevating product quality.

## Data availability statement

The datasets presented in this study can be found in online repositories. The names of the repository/repositories and accession number(s) can be found in the article/Supplementary material.

## Author contributions

XW: Conceptualization, Formal analysis, Investigation, Methodology, Software, Writing – original draft. SY: Data curation, Writing – original draft. QG: Investigation, Visualization, Writing – original draft. YD: Data curation, Writing – original draft. LT: Investigation, Visualization, Writing – original draft. LW: Investigation, Visualization, Writing – original draft. HY: Resources, Supervision, Writing – original draft. LY: Software, Validation, Writing – original draft. XH: Software, Validation, Writing – original draft. PL: Conceptualization, Funding acquisition, Resources, Supervision, Writing – original draft. LZ: Conceptualization, Funding acquisition, Resources, Supervision, Writing – review & editing.

## Footnotes

^1^ http://www.mothur.org/wiki/Calculators

^2^ http://huttenhower.sph.harvard.edu/LEfSe

^3^ http://www.hmdb.ca/

^4^ https://metlin.scripps.edu/

^5^ https://cloud.majorbio.com
