# Supplementary figures and images for: Multi-omics reveals the phyllosphere microbial community and material transformations in cigars
Source: Front Microbiol. 2024 Jul 31;15:1436382. doi: 10.3389/fmicb.2024.1436382 (PMC11322134; doi:10.3389/fmicb.2024.1436382)

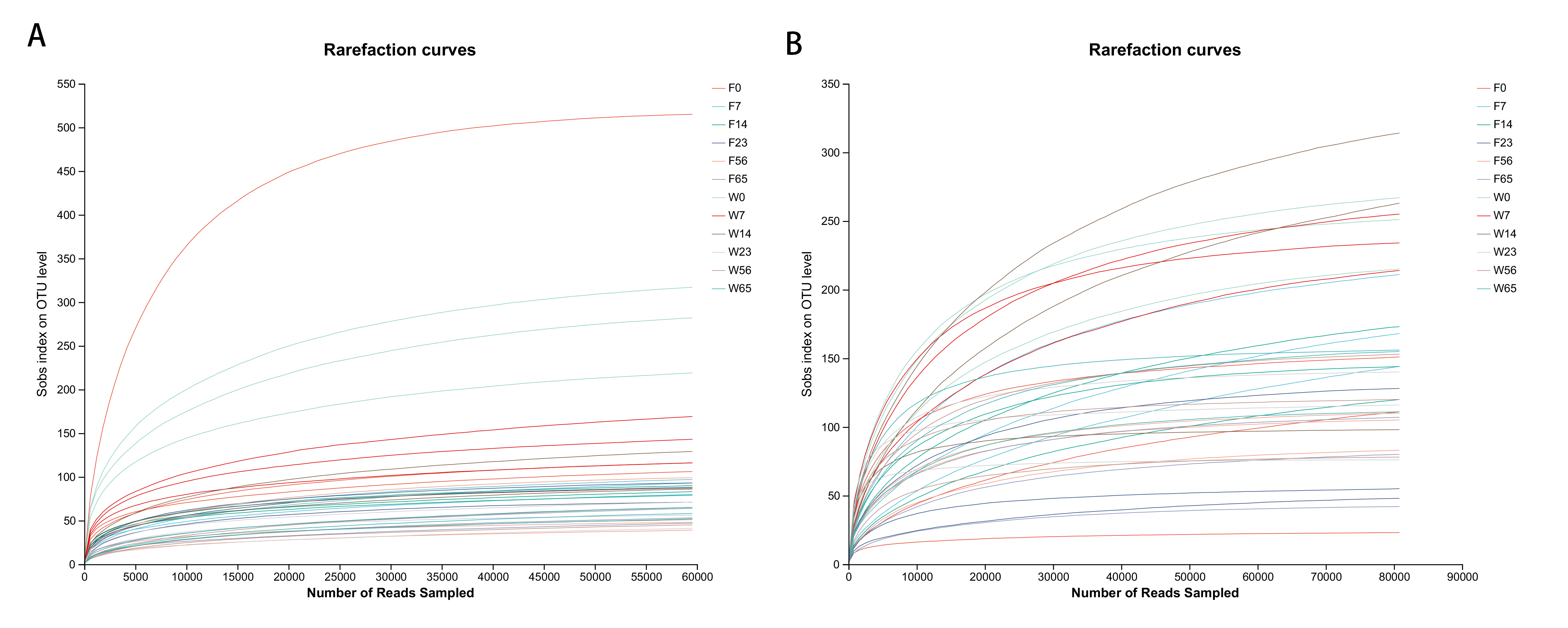

Supplement: Supplementary file 1 [file Image_1.TIF]

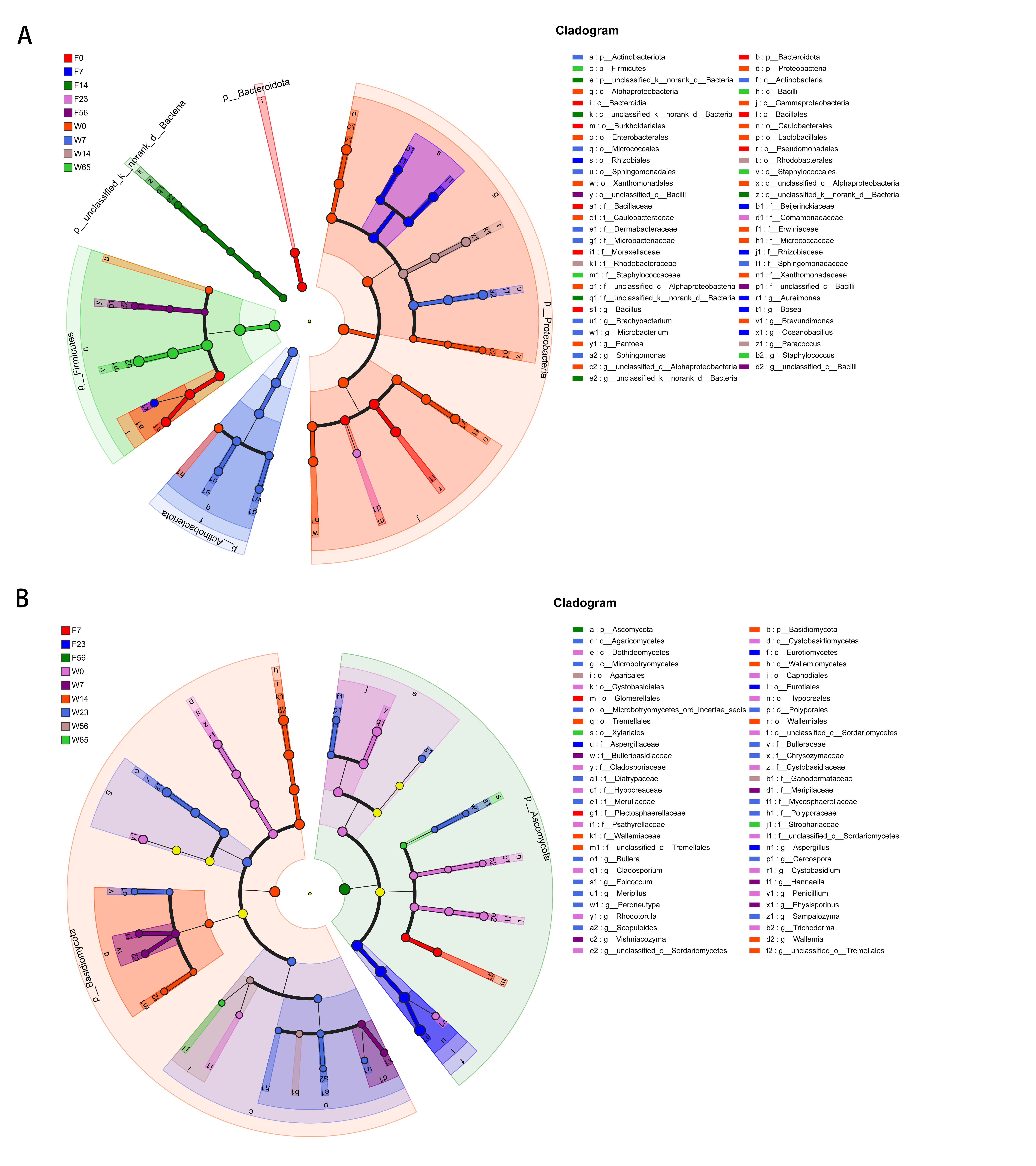

Supplement: Supplementary file 2 [file Image_2.TIF]

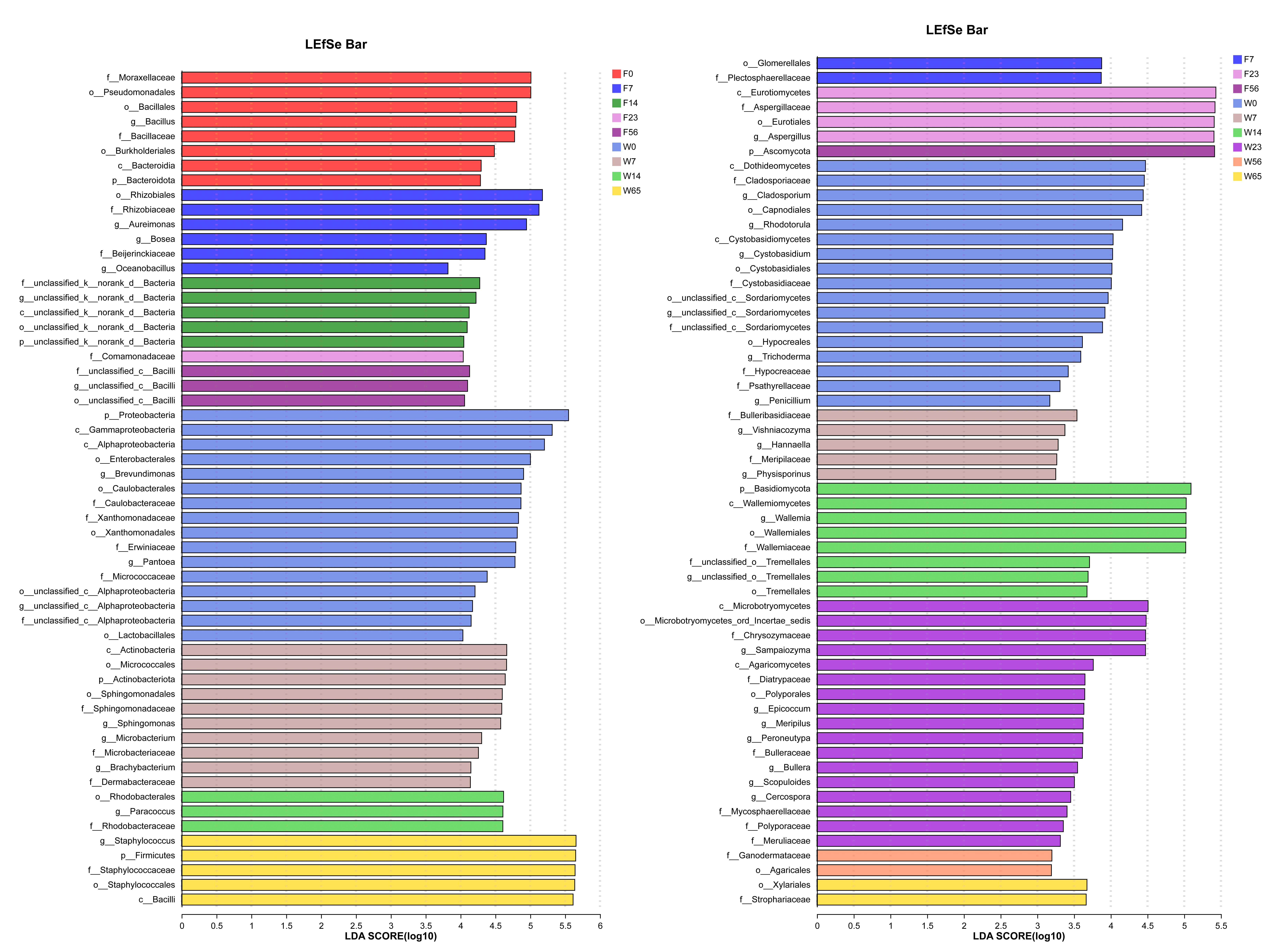

Supplement: Supplementary file 3 [file Image_3.TIF]

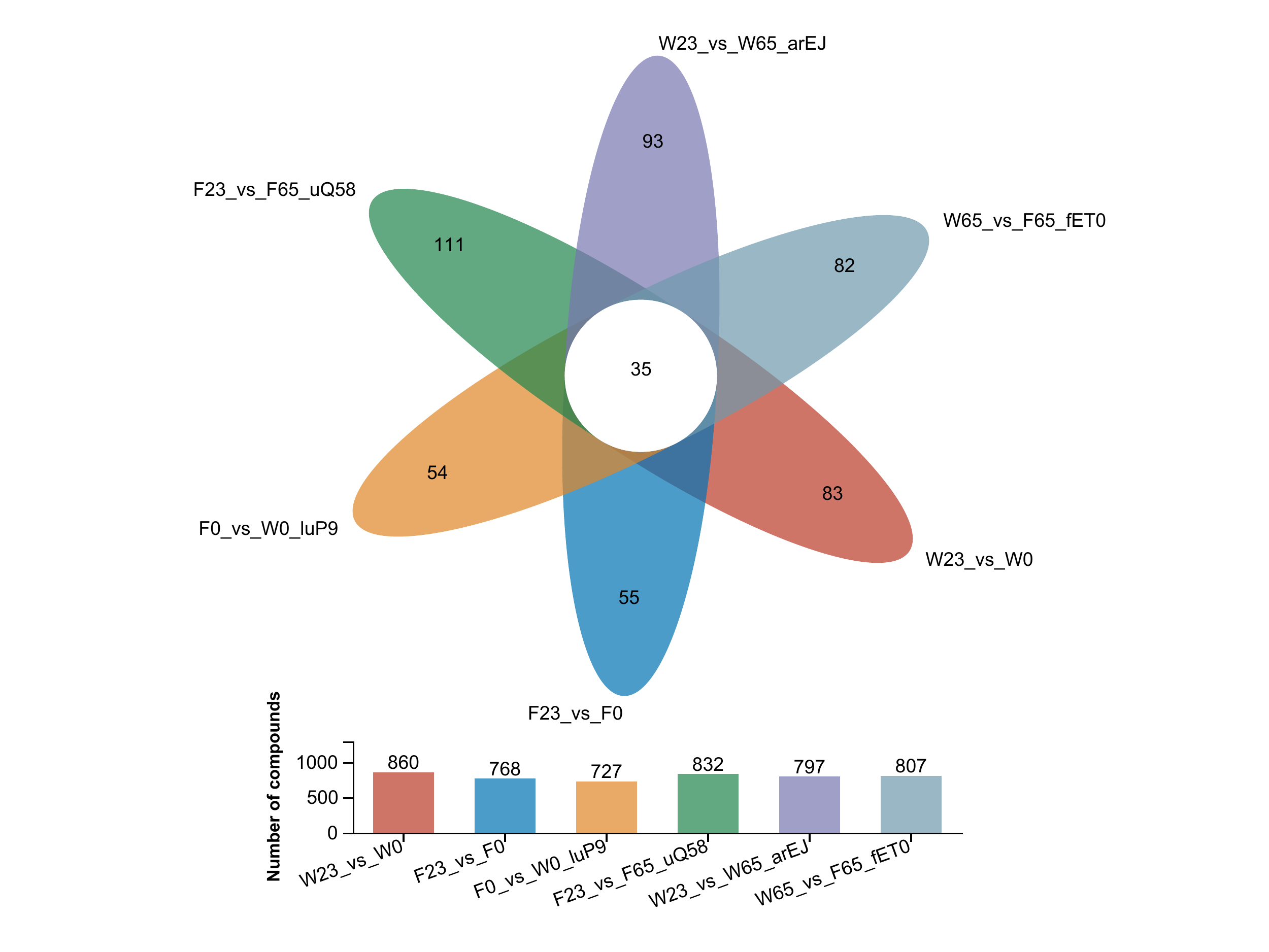

Supplement: Supplementary file 4 [file Image_4.TIF]
